# Oxidative Stress in Military Missions—Impact and Management Strategies: A Narrative Analysis

**DOI:** 10.3390/life14050567

**Published:** 2024-04-27

**Authors:** Dumitru Radulescu, Florina-Diana Mihai, Major Emil-Tiberius Trasca, Elena-Irina Caluianu, Captain Dan Marian Calafeteanu, Patricia-Mihaela Radulescu, Razvan Mercut, Eleonora Daniela Ciupeanu-Calugaru, Georgiana-Andreea Marinescu, Cristian-Adrian Siloşi, Colonel Claudiu Eduard Nistor, Suzana Danoiu

**Affiliations:** 1 Department of Surgery, The Military Emergency Clinical Hospital ‘Dr. Stefan Odobleja’ Craiova, 200749 Craiova, Romania; dr_radulescu_dumitru@yahoo.com (D.R.); irina.caluianu@umfcv.ro (E.-I.C.); paty_miha@yahoo.com (P.-M.R.); marinescu_georgiana.andreea@yahoo.com (G.-A.M.); 2 Doctoral School, University of Medicine and Pharmacy of Craiova, 2 Petru Rares Street, 200349 Craiova, Romania; cristian.silosi@umfcv.ro; 3 Department of Ortopedics, The Military Emergency Clinical Hospital ‘Dr. Stefan Odobleja’ Craiova, 200749 Craiova, Romania; danutcalafeteanu@yahoo.com; 4 Department of Plastic and Reconstructive Surgery, University of Medicine and Pharmacy of Craiova, 200349 Craiova, Romania; mercut.razvan@umfcv.ro; 5 Department of Biology and Environmental Engineering, University of Craiova, 200585 Craiova, Romania; ciupeanudaniela@gmail.com; 6 Department of Thoracis Surgery, Carol Davila University of Medicine and Pharmacy, 020021 Bucharest, Romania; ncd58@yahoo.com; 7 Department of Pathophysiology, University of Medicine and Pharmacy of Craiova, 200349 Craiova, Romania; suzanadanoiu@yahoo.com

**Keywords:** military mission, oxidative stress, ketogenic diet, physical exercise

## Abstract

This narrative review comprehensively examines the impact of oxidative stress on military personnel, highlighting the crucial role of physical exercise and tailored diets, particularly the ketogenic diet, in minimizing this stress. Through a meticulous analysis of the recent literature, the study emphasizes how regular physical exercise not only enhances cardiovascular, cognitive, and musculoskeletal health but is also essential in neutralizing the effects of oxidative stress, thereby improving endurance and performance during long-term missions. Furthermore, the implementation of the ketogenic diet provides an efficient and consistent energy source through ketone bodies, tailored to the specific energy requirements of military activities, and significantly contributes to the reduction in reactive oxygen species production, thus protecting against cellular deterioration under extreme stress. The study also underlines the importance of integrating advanced technologies, such as wearable devices and smart sensors that allow for the precise and real-time monitoring of oxidative stress and physiological responses, thus facilitating the customization of training and nutritional regimes. Observations from this review emphasize significant variability among individuals in responses to oxidative stress, highlighting the need for a personalized approach in formulating intervention strategies. It is crucial to develop and implement well-monitored, personalized supplementation protocols to ensure that each member of the military personnel receives a regimen tailored to their specific needs, thereby maximizing the effectiveness of measures to combat oxidative stress. This analysis makes a valuable contribution to the specialized literature, proposing a detailed framework for addressing oxidative stress in the armed forces and opening new directions for future research with the aim of optimizing clinical practices and improving the health and performance of military personnel under stress and specific challenges of the military field.

## 1. Introduction

### 1.1. Contextualization

A military career is characterized by a rigorous and challenging lifestyle, demanding from military personnel a constant commitment and a remarkable capacity for adaptation. This reality persists regardless of the nature of the missions or the geographical context, subjecting military personnel to a broad spectrum of conditions that can test the limits of human endurance, both physically and psychologically. Factors such as frequently hostile operating environments and the continuous pressure of responsibilities related to defense security and peace promotion constitute fundamental aspects of military life.

An essential element of this challenging framework is the exposure to oxidative stress during military training. Systematic reviews indicate that oxidative stress in these contexts is associated with significant changes in hormone levels and biomarkers for cellular injury, resulting from intense training exercises that involve high energy expenditure, combined with dietary and sleep restrictions [1]. These findings underscore the critical need to recognize and manage oxidative stress as a vital component of training and recovery among military personnel.

Furthermore, studies focused on assessing oxidative stress, muscle damage, and psychomotor skills among special forces during military survival training indicate that, although intense training did not directly cause significant oxidative stress or muscle damage, and even improved psychomotor abilities, post-training changes in strength may suggest a potential deterioration in motor control [2]. These exacerbated stress conditions can negatively impact operational performance and are predisposing factors for the development of mental health disorders, including anxiety and post-traumatic stress disorder (PTSD) [3]. PTSD, in particular, represents one of the most debilitating psychiatric conditions encountered among Romanian military personnel and veterans, associated with various comorbidities that can worsen physical and mental health over the extended post-mission periods. Sleep-related issues, including insufficiency and poor quality, amplify vulnerability to physical and mental disorders, underscoring the necessity for effective stress management strategies and the prevention of oxidative stress among military personnel [4].

### 1.2. Central Issue 

The central issue of this analysis focuses on oxidative stress, defined as an imbalance between the production of reactive oxygen species (ROS) and the body’s antioxidant defense system’s capacity [5]. This condition is exacerbated in the military context, where exposure to extreme conditions, including specific pollutants and heavy metals resulting from activities and armed conflict, significantly contributes to the oxidative burden.

Situated at the core of metabolic processes, in mitochondria and the endoplasmic reticulum, oxidative metabolism is responsible for generating by-products, including free radicals and other reactive oxygen and nitrogen molecules (ROS/RNS), which, under conditions of imbalance, contribute to oxidative or nitro-oxidative stress [6,7]. This condition is implicated in the pathogenesis of a wide spectrum of chronic diseases, from cancer and diabetes to neurodegenerative and cardiovascular diseases.

The peculiarities of military activities, including exposure to heavy metals through ammunition spills and the use of depleted uranium, heighten oxidative stress risks due to the toxic effects of these metals on human health, including neurological, cardiovascular, and fertility impairments [8]. Epidemiological studies have highlighted the association between professional exposure to uranium and an increased risk of lung cancer, with the kidneys, lungs, brain, and liver being the primary target organs. Additionally, the inhalation of toxic vapors and exposure to sarin and cyclosarin in military conflicts have been linked to respiratory difficulties and other severe adverse health effects [9].

Military personnel are often exposed to highly detrimental environmental conditions, including dust storm particles and emissions from burn pits, with potentially dire consequences on the respiratory system [8]. In this context, the role of heavy metals, both essential and non-essential, becomes a major concern. While metals like copper are vital for the functioning of enzymes that participate in the oxidative stress response, exposure to non-essential heavy metals such as cadmium and lead can have dangerous effects even at low doses [10].

This issue is exacerbated by air pollution resulting from military operations, including recent ones in the Russia–Ukraine conflict, underscoring the risks of inhaling pollutants and the need for further studies to assess the long-term impact of these exposures on human health [11]. Zinc, for example, can become toxic in cases of excessive accumulation, leading to symptoms such as fever, breathing difficulties, and nausea [12].

In the specific context of the military environment, the challenges posed by oxidative stress, along with exposure to heavy metals and various other toxins, constitute a significant concern for the health status of personnel [7]. This underscores the imperative for conducting detailed epidemiological assessments, biomonitoring procedures, and dedicated laboratory investigations, with the aim of accurately decoding the underlying mechanisms of toxicity and developing effective risk mitigation methods. Addressing this complexity is essential in ensuring the health protection of military mission participants against a broad range of environmental and biological hazards.

### 1.3. Purpose of the Review

The purpose of this narrative review is to analyze in detail the impact of oxidative stress on military missions, with a particular focus on identifying the mechanisms through which it affects the health and performance of military personnel. It also aims to evaluate and synthesize effective management and intervention strategies designed to mitigate the negative impact of oxidative stress. Through this investigation, we aim to aggregate and evaluate the existing specialized literature, paying special attention to studies that directly address the connection between the specific conditions of military activities and oxidative stress. This endeavor includes assessing exposure to environmental factors characteristic of the military environment, which can intensify oxidative stress.

## 2. Materials and Methods

**Figure 1:**
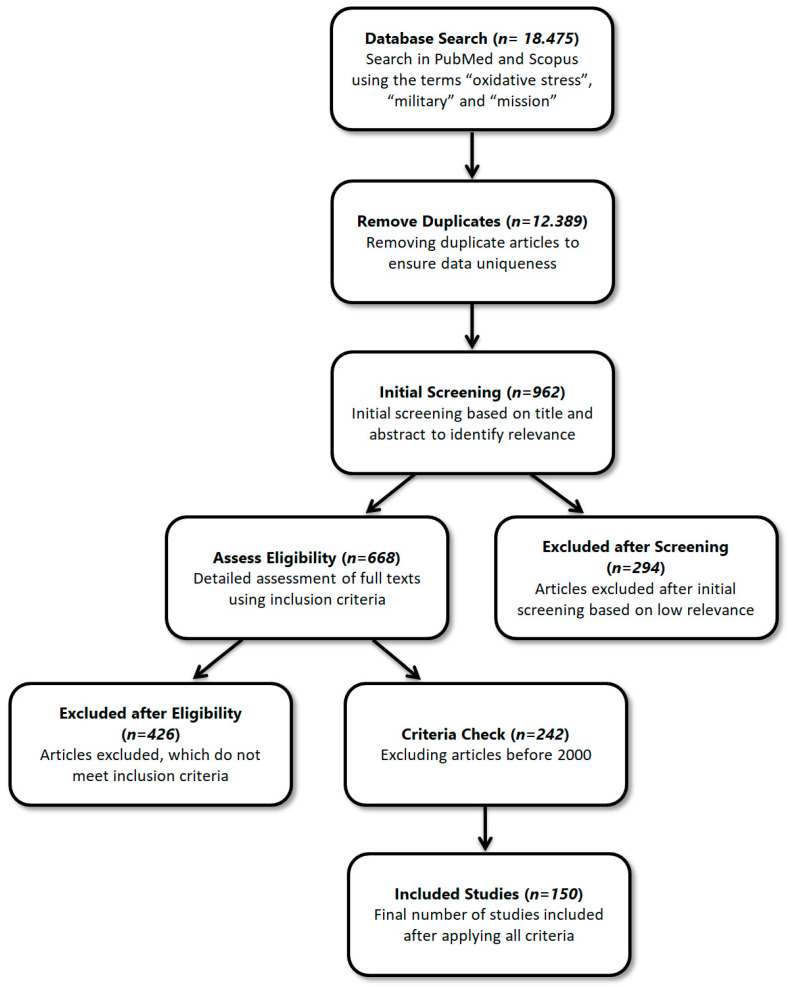
Article Selection Flowchart.

The methodology adopted in this review involved an exploratory search of relevant scientific publications through the databases PubMed and Scopus, using key terms such as “oxidative stress”, “military”, and “mission”. To ensure the relevance and high quality of the studies included in the review, strict inclusion criteria were applied. Thus, conference presentations, editorials, publications without open access, and works published before the year 2000 were excluded to ensure that the analysis is based on updated and accessible information (Figure 1).

This methodical approach allows us not only to identify and evaluate the impact of oxidative stress on military personnel but also to explore available prevention and treatment strategies, such as dietary interventions, antioxidant supplementation, and lifestyle modifications that could contribute to reducing associated risks. Additionally, we recognize the importance of further research in this field to develop more effective and personalized oxidative stress management methods for military personnel, considering the specific challenges and conditions they face.

Through this narrative analysis, we aim to make a significant contribution to understanding the phenomenon of oxidative stress in the context of military missions and to the development of evidence-based management strategies, thus laying a solid foundation for optimizing the health and performance of military personnel in extreme stress situations.

### Research Questions and Hypotheses

The in-depth exploration of oxidative stress within military missions, as presented in the narrative review, directs our attention to a set of fundamental research questions and hypotheses deserving of thorough investigation. These are designed to address the interindividual variability in response to exercise, the characteristics of an optimal diet to maximize adaptation to oxidative stress, the synergy between exercise and nutrition, and the need for developing personalized strategies. Each of these areas opens the path to deepening our knowledge and optimizing interventions in the unique context of the military environment.

These research questions form the foundation upon which future studies can build to provide personalized and effective solutions in the management of oxidative stress, with the potential to revolutionize preventive and therapeutic approaches for military personnel. Therefore, the discussion and hypotheses generated are summarized in the following table, which serves as an essential guide for directing future research in this critical field (Table 1).

## 3. Theoretical Foundations

### 3.1. Physical Exercise and Oxidative Stress

Physical exercise (EXR) is widely acknowledged for its extensive benefits on human health, positively influencing multiple body systems and thereby reducing the risk of mortality associated with various chronic diseases, including cardiovascular diseases, cancer, metabolic disorders, and conditions of the central nervous system [13]. This crucial health intervention significantly improves cardiovascular capacity, cognitive function, immune activity, endocrine balance, and musculoskeletal health, providing a solid foundation for promoting well-being in individuals of all ages.

The beneficial effects of EXR are partly mediated through the body’s adaptation to redox homeostasis, neutralizing the sudden increase in reactive oxygen species [ROS]—a key element in the pathogenesis of many chronic diseases. Furthermore, physical exercise initiates the release of a variety of humoral factors, such as proteins, microRNAs (miR), and DNA, transported via extracellular vesicles (EVs). These EVs exhibit changes in their load in response to oxidative stress and physical activity, indicating a pathway through which EXR can positively influence antioxidant enzyme pathways and mitigate the oxidative stress environment [13].

Divided into endurance and resistance exercise categories, EXR represents a source of voluntary body movement generated by skeletal muscles that leads to energy consumption. This type of activity demands the body to quickly adapt to increased metabolic and physiological requirements, demonstrating the flexibility and responsiveness of the cardiovascular and muscular system to effort [14].

Following exercise, cardiomyocytes and skeletal muscle cells undergo adaptive hypertrophy, thereby improving oxygen utilization capacity and reflecting an increase in cardiorespiratory fitness (CRF). This adaptation is measured through the increase in maximal oxygen consumption (VO2max), which can rise by 40–50% in response to EXR, offering significant benefits for cardiovascular health and serving as prevention for individuals at increased risk of developing cardiovascular diseases and for patients with pathological conditions such as heart failure [15,16,17].

Beyond cardiovascular improvements, EXR has a remarkable positive impact on cognitive function, enhancing working memory, attention, processing speed, and inhibitory control. These cognitive benefits, affirmed by multiple studies, are attributed to the increase in aerobic capacity induced by exercise, which favors cognitive improvement through enhanced synaptic plasticity, highlighting the essential role of EXR in supporting mental health and preventing cognitive decline [18,19].

High-intensity physical exercises have been shown to modulate the response of the hypothalamic–pituitary–adrenal (HPA) axis, crucial in stress regulation. This modulation includes inhibiting cortisol release, a critical hormone in the stress response that, under normal conditions, mobilizes glucose to provide energy to the body. Inhibiting this mechanism at the end of exercise activities suggests a protective effect against additional stress, thus underlining the therapeutic role of exercise in stress management and in improving metabolic health, including in the context of disorders related to obesity and insulin resistance, precursors of type 2 diabetes [20,21].

Furthermore, physical exercise stimulates immune function through the release of anti-inflammatory cytokines, with variations determined by the intensity and duration of the exercise [22]. This ability of exercise to regulate the immune response and promote an anti-inflammatory state underscores its importance in maintaining health and preventing diseases.

Exercise also contributes to improving muscular endurance and increasing cells’ capacity to resist oxidative stress. By enhancing the contractile properties of muscles and promoting angiogenesis, physical exercise supports positive muscular adaptations, essential for physical performance and resilience to injuries [23,24].

The increased energy demand during exercise is met by optimizing mitochondrial function, which, under the influence of exercise, enhances its biogenesis and cellular metabolism. This adaptation increases ATP availability, thereby supporting performance and endurance to effort [25,26]. In this context, mitochondria, along with NADPH oxidase and xanthine oxidase, serve as endogenous sources of reactive oxygen species (ROS), essential for cellular signaling and maintaining homeostasis [27].

The reactive oxygen species generated during exercise can also originate from external sources to muscle cells, including immune cells and the endothelium. The increase in ROS concentration can induce oxidative stress, negatively affecting cellular function and metabolic balance [28]. However, regular exercise induces adaptations to redox-sensitive pathways, known as “redox homeostasis”, which contribute to protection against the harmful effects of oxidative stress [29].

These adaptations include the activation of complex signaling pathways and antioxidant defense mechanisms, among them the increased transcription of genes encoding key antioxidant enzymes, such as catalase, superoxide dismutase, heme oxygenase-1, and NAD(P)H quinone dehydrogenase 1, crucial in neutralizing ROS [30,31]. Nuclear factor erythroid 2 (NRF2), a central element in the regulation of antioxidant pathways, is activated by exercise, promoting the increased expression of antioxidant enzymes and, therefore, a beneficial adaptation to oxidative stress [32].

Recent studies have revealed that training periods in athletes are associated with an increase in the body’s antioxidant capacity and activation of detoxification processes, suggesting a positive impact of physical exercises on redox homeostasis [33]. For instance, a study conducted on a group of elderly and overweight individuals who performed aerobic dance training demonstrated a significant decrease in serum levels of malondialdehyde (MDA), a well-known marker of oxidative stress [34].

Research indicates that regular physical activity promotes redox homeostasis through the activation of complex antioxidant pathways, leading to a decrease in oxidative stress markers [13]. This adaptation to redox homeostasis has the potential to bring various health benefits in different health contexts and diseases.

Tissue crosstalk, essential for triggering adaptive effects in multiple tissues, is not yet fully understood in terms of the specific molecular mechanisms that facilitate communication between organs and coordinate the positive effects of physical exercises [35]. However, it is known that, during exercise, skeletal muscles and other cell types can release peptides and nucleic acids that can be taken up by other organs.

These effort-induced factors, collectively termed “exerkines”, mediate systemic adaptations to exercise [36]. Unlike myokines and adipokines, which are peptides and miRNAs produced and secreted by skeletal muscles and fat deposits, “exerkines” include all exercise-induced humoral factors (peptides and RNA species) that are expressed, produced, and secreted by all tissues and organs into the bloodstream to promote inter-organ communication and enhance the systemic benefits of physical exercises [37].

It is important to note that proteins without a canonical secretion-targeting sequence, proteins whose secretion depends on external stimuli, and molecules that may be unstable in the extracellular environment are preferentially secreted through extracellular vesicles (EVs) [38]. EVs are small membranous structures secreted by various cells as information vehicles and can transport diverse biomolecules, including proteins, lipids, and nucleic acids [39]. Recently, it has been shown that EVs play a significant role in the beneficial effects mediated by physical exercise [40].

Produced extracellular vesicles (EVs) are introduced into the biological fluid (peripheral blood, saliva, and amniotic fluid) to reach their specific target where they release their load [41]. EVs are typically defined by specific surface markers. In this review, we highlight the role of EVs in the response to oxidative stress and the modulation of redox homeostasis, with a particular focus on the role of EXR. The latter has been shown to have beneficial effects on various organs and tissues by attenuating oxidative stress and promoting the adaptation of redox homeostasis. This adaptive process involves the activation of complex molecular pathways, including transcription factors (NRF2), antioxidant enzymes [AOEs], and non-enzymatic molecules, resulting in a reduction in oxidative stress levels.

The collective evidence from these studies strongly supports the positive impact of EXR on redox homeostasis at both the cellular and molecular levels. The EV load, modulated by EXR, appears to enhance antioxidant capacity, reduce oxidative stress, and activate detoxification processes, all contributing to maintaining a balanced redox state. Understanding the effects of the reduced oxidative stress environment mediated by regular EXR is crucial for preventing various diseases and aging processes. Indeed, an increase in ROS can lead to a reduction in nitric oxide (NO) availability, causing vasoconstriction and promoting hypertension [42].

Furthermore, the imbalance in redox homeostasis is a key hallmark of many diseases, such as cancer, Alzheimer’s, and metabolic disorders. Therefore, understanding the effects of exercise on redox homeostasis may have significant implications for optimizing exercise interventions, as well as for promoting general health and well-being. Various types of exercises, such as HIIT, have shown considerable effects on redox homeostasis in various human cohorts [43].

The complex interaction between EXR, EVs, and their load is highlighted. The evidence reported in this review suggests that EVs released into circulation during physical activity have an interesting antioxidant role, warranting further investigation. Additional research is needed to elucidate the specific mechanisms underlying these effects and to explore the potential therapeutic applications of the effort-induced modulation of EV load. Furthermore, these studies can help us discover additional interconnections and expand our understanding of the complex relationship between EVs and oxidative stress and how interventions, such as EXR, can lead to a more effective response.

Regular exercise enhances endurance and strength, which are essential for meeting physical demands in combat situations or extended missions. Additionally, physical exercise plays a crucial role in maintaining mental health by reducing the impact of psychological stress encountered in combat conditions [44]. Weight management, improving insulin sensitivity, and regulating glucose levels are vital aspects in preventing and managing metabolic syndrome and type 2 diabetes. Studies have shown that physical fitness programs can significantly reduce risk factors for metabolic syndrome among military personnel [45].

Structured workouts and organized sports activities, forms of group physical exercise, can significantly improve communication and cooperation among unit members. These activities not only support physical fitness but also create opportunities for team members to work together towards common goals, essential for unit cohesion [46]. Increased cohesion in military units, stimulated through joint exercises, can provide significant protection against the adverse effects of military stress. Soldiers in units with strong cohesion report improved physical and psychological well-being and greater satisfaction with their military careers compared to those in less cohesive units [47].

Enhancing cohesion during Basic Combat Training is associated with reduced psychological stress and improved stress management, contributing to the promotion of positive morale [48]. In the infantry, the ability to perform long marches under full gear is crucial, and endurance training is essential. A study highlighted the importance of integrating endurance training with strength training to optimize soldiers’ overall physical performance, demonstrating the superiority of these combined training methods [49].

Special units, which often perform tasks requiring rapid strength and agility, can greatly benefit from training programs that emphasize explosive strength and speed. A recent study examined the effects of linear periodized resistance training on cadets at a naval academy, revealing significant improvements in muscular strength, agility, and reaction time, underlining the efficacy of this type of training for improving specific conditioning needs [50].

Wireless distributed sensor systems, such as body sensor networks, play a crucial role in the continuous monitoring of stress and physiological responses during intense training, providing a detailed perspective on the impact of stress on the body [51]. Modern technology, including wearable devices and biometric sensors, enables the real-time monitoring of oxidative stress biomarkers and other physiological parameters [52]. This technological approach facilitates the dynamic adaptation of training programs, optimizing both physical performance and oxidative stress management, allowing military trainers and doctors to customize workouts to maximize benefits and minimize risks [51].

Improving physical mobility and functionality: A recent study on older veterans with PTSD demonstrated that participation in a physical exercise program significantly enhances physical function and reduces clinical risk factors for chronic diseases. Participants in the exercise group showed notable improvements in aerobic endurance and physical performance, highlighting long-term positive effects on health and quality of life [53]. For a summary of the relevant studies and findings discussed in this chapter, see Table 2.

### 3.2. Diet and Oxidative Stress

The ketogenic diet, characterized by high fat, low to moderate protein, and low carbohydrate intake, adheres to a macronutrient ratio of approximately 3–4:1, with the distribution being 90% fats, 6% proteins, and 4% carbohydrates. This nutritional approach was initially developed as a non-pharmacological therapy for epilepsy in 1923 [54]. The central mechanism of the ketogenic diet aims to optimize mitochondrial metabolism, demonstrating a link between its practice and the improvement of mitochondrial function, as well as a significant reduction in oxidative stress [55].

At the heart of the ketogenic diet is the production of ketone bodies, such as beta-hydroxybutyrate (bHB) and acetoacetate (AA), resulting from enhanced fatty acid oxidation in the liver. These precursors to acetyl CoA mark the initial stage of the citric acid cycle. Beta-hydroxybutyrate, the most studied ketone body, is recognized for its ability to reduce the production of reactive oxygen species (ROS), enhance mitochondrial respiration, and stimulate the cell’s endogenous antioxidant system [54].

Oxidative stress is defined as a chemical imbalance between the production of free radicals and the antioxidant system’s capacity to neutralize these reactive compounds. This imbalance is characterized by the excessive production of free radicals and reactive oxygen species (ROS) as cells use oxygen to generate energy, with antioxidants serving to counteract and protect against these harmful species [56]. The primary endogenous sources of free radicals include mitochondria, while sunlight exposure and smoking are relevant external sources.

Damage to biomolecules and cells, including deoxyribonucleic acid (DNA), lipids, and proteins, is a direct consequence of the imbalance between antioxidants and free radicals, leading to sustained oxidative stress and potential critical injuries to cellular structure. This can facilitate the onset of somatic mutations and neoplastic transmutations due to long-term ROS production in a state of prolonged oxidative stress.

Oxidative stress results from the interaction between the production of free radicals and the body’s antioxidant defense mechanisms, leading to excessive production of reactive oxygen species. This imbalance leads to oxidative stress and triggers a wide range of diseases [57]. Thus, the ketogenic diet, by promoting an efficient energy metabolism and reducing oxidative stress, offers an interesting framework for studying dietary interventions in the context of managing oxidative stress, highlighting its therapeutic potential in various pathological states and in enhancing general health.

The pathogenesis of oxidative stress is closely linked to inflammation, which can have multiple sources, including microbial and viral infections, exposure to toxic chemicals, autoimmune diseases, chronic obesity, and alcohol and tobacco consumption. It is observed that the risk of developing cancer increases as inflammation persists over long periods [56].

Inflammation can be classified into two phases: acute and chronic. Acute inflammation represents the initial phase, characterized by a short duration and often beneficial to the body, initiated by the activation of the immune system. On the other hand, chronic inflammation is long-lasting and can increase one’s susceptibility to various chronic diseases, including cancer [58].

During the inflammatory process, mast cells and leukocytes are recruited to the site of injury, leading to a “respiratory burst” by increasing oxygen uptake and, consequently, the release and increased accumulation of reactive oxygen species (ROS) in the affected area [59].

Reactive oxygen species (ROS) are naturally produced as a result of cellular metabolism; however, oxidative stress is described as a pathological condition when the balance between the production of oxidants and detoxification processes favors a pro-oxidant state, overwhelming antioxidant defense and resulting in the accumulation of reactive species that can damage nucleic acids, proteins, and membrane lipids [60].

The DNA repair system contributes to maintaining the balance between the generation and elimination of ROS. In the context of cellular protection against radicals, antioxidants prove to be more specific and efficient. These antioxidants can be of endogenous or exogenous origin, enzymatic or non-enzymatic, forming a complex and multifunctional antioxidant system [56].

The action mechanism of the ketogenic diet leads to a decrease in blood glucose levels and an increase in blood ketone levels, thus contributing to the inhibition of tumor development in both humans and animals. This effect is partly due to the fact that ketone body metabolism protects cells against oxidative damage by inhibiting the production of reactive oxygen species (ROS) and by enhancing the cell’s endogenous antioxidant capacity [61]. A study on healthy women highlighted that adopting a ketogenic diet for 14 days, involving dietary restrictions, resulted in weight loss and a marked improvement in total antioxidant status, without inducing oxidative stress in the blood [62].

Although this study utilized the ketogenic diet without a control group, making it challenging to determine whether the antioxidant effects were directly attributed to the diet or the weight loss resulting from caloric restriction, it was observed that the ketogenic diet favored the production of the antioxidant glutathione (GSH) [63]. The beneficial effects of the ketogenic diet also extend to the reduction in inflammation and thermal nociception, considering its ability to limit the production of reactive oxygen species (ROS) and to improve the expression and activity of mitochondrial uncoupling proteins [63].

A crucial aspect of the ketogenic diet is related to the improvement of mitochondrial function, facilitated by ketogenic metabolic activity and the reduction in oxidative stress [56]. Specifically, the activity of beta-hydroxybutyrate (β-HB), the most studied ketone body, is known for reducing ROS production [64]. Β-Hydroxybutyrate also stimulates mitochondrial respiration by activating the nuclear factor erythroid 2-related factor [Nrf2], which in turn initiates the cell’s endogenous antioxidant system. Nrf2 plays an essential role in promoting the synthesis of vital enzymes for regenerating active endogenous antioxidants, such as glutathione reductase, thioredoxin, and peroxiredoxin [65].

Furthermore, β-HB acts as an endogenous inhibitor of class I and IIa histone deacetylases (HDACs), facilitating the transcription of genes responsible for detoxification, including catalase, mitochondrial superoxide dismutase (mn-SOD), and metallothionein, providing protection against oxidative stress [66]. The ketogenic diet also modulates the intracellular NAD+/NADH ratio, recognized for its protective effects against ROS, constituting another mechanism through which the ketogenic diet exerts a protective effect against oxidative stress [67].

By limiting glucose availability for glycolysis and, thus, the synthesis of pyruvate and glucose-6-phosphate, which could fuel the pentose phosphate pathway for the production of essential NADPH in reducing hydroperoxides, the ketogenic diet restricts glucose metabolism. This restriction encourages cells to generate energy through mitochondrial lipid metabolism, thereby forcing cancer cells to experience oxidative stress [54].

Studies have highlighted the ketogenic diet’s potential to improve mitochondrial antioxidant status, providing protection to mitochondrial DNA (mtDNA) against oxidative damage. mtDNA, being highly sensitive to reactive oxygen species (ROS), can benefit from protection in the context of the ketogenic diet, as demonstrated in an animal study led by Yang et al. In this study, rats fed a ketogenic diet did not show significant mtDNA damage, while in the control group, the frequency of oxidative lesions was significantly higher at all time points assessed (*p* < 0.0001) [68].

Moreover, ketogenic diets have been associated with decreased levels of DNA damage and rapid changes in the activity of PARP-1 enzymes and sirtuin, suggesting that these diets could provide effective protection to healthy cells against oxidative and metabolic damage [69]. It was observed that the ketogenic diet exerts specific effects on malignant brain tissue, influencing the expression of genes related to ROS level regulation [70].

The specific ketone bodies of the ketogenic diet, beta-hydroxybutyrate and acetoacetate, contribute to reducing ROS production, thus demonstrating the diet’s ability to modulate oxidative stress in a beneficial manner [71].

This diet also has a positive impact on antioxidant capacity, promoting the enhanced biosynthesis of the antioxidant glutathione (GSH). The findings obtained by Stafford et al. indicate that the ketogenic diet could be considered a promising strategy for the prevention and treatment of certain pathological conditions, including cancer. However, due to current limitations regarding the availability of controlled studies exploring the effects of the ketogenic diet on oxidative stress and cancer, further research is essential to solidify the evidence base on the anticancer benefits of this dietary approach [71].

Oxidative stress levels in the body are influenced by both individual and environmental factors, where diet plays a significant role and represents an easily modifiable aspect [72]. The importance of diet in the development and progression of chronic diseases is underscored by its direct association with oxidative stress, a common pathogenic mechanism of these diseases [73]. A study conducted by Kong et al. on 335 Chinese citizens, aged over 60 years and without major conditions or recent treatments that could influence the measurement of oxidative stress, explored this link [74], and the participants’ dietary diversity scores showed a preference for cereals over fish, reflecting similar findings from other regional research [75].

The analysis highlighted that a higher-quality diet is associated with improved Total Antioxidant Capacity (T-AOC), suggesting that healthier eating can enhance antioxidant levels in the body. The results confirm that a high-quality diet corresponds with better levels of oxidative stress markers. Participants who followed a higher-quality diet presented a higher T-AOC, indicating a better balance of oxidative stress. This underscores the importance of dietary diversity and quality in managing oxidative stress, especially among the elderly population [74].

The connection between diet and oxidative stress is strengthened by findings such as the beneficial effects of cherry juice on inflammation and oxidative stress biomarkers, highlighting the diet’s capacity to modulate these processes [76]. Previous studies have indicated oxidative stress markers as early indicators of the risk for chronic diseases, and the Mediterranean diet has been associated with a reduction in this risk, unlike diets high in fats [73,77].

Research conducted on mice has indicated that the ketogenic diet may reduce mid-life mortality and enhance memory performance in old age through mechanisms including decreased insulin levels and protein synthesis, as well as increased mitochondrial efficiency. These effects could have significant implications for human resilience in survival scenarios [78]. Another study on mice demonstrated that the ketogenic diet improves mitochondrial function and reduces oxidative stress, contributing to better energy efficiency and the maintenance of muscular integrity under conditions of prolonged physical stress [79].

The ketogenic diet (KD) has been successfully applied among military personnel, showing promising results in managing energy and endurance—crucial elements for long-term missions or extreme conditions. LaFountain and colleagues observed that military personnel who adopted a KD experienced significant reductions in weight, body fat, and particularly visceral fat, without compromising the essential physical performance adaptations necessary for their training. These findings suggest that KD could improve overall health and readiness without negatively affecting the physical capabilities required for fulfilling their roles [80].

KD supports the maintenance of physical performance and cognitive function in challenging conditions through its stable and efficient energy source derived from ketone bodies. This is particularly valuable in situations where traditional high-carbohydrate diets may be impractical [81]. In scenarios of limited access to food, simplifying the diet by focusing on fat consumption can facilitate the management of food resources, making the ketogenic diet a practical and sustainable option. For a summary of the relevant studies and findings discussed in this chapter, see Table 3.

### 3.3. Antioxidants

Over the years, the perception of antioxidant supplements in the field of nutrition associated with physical exercise has significantly fluctuated, spanning a wide spectrum from being considered essential to being labeled as potentially harmful. Initially, about 35 years ago, in the context of discoveries regarding the production of reactive oxygen, nitrogen, and sulfur species during physical activity, antioxidant supplements were seen as a crucial element for combating the harmful effects of exercise-induced oxidative stress [82]. However, over the last decade, this opinion has radically changed, with numerous studies and review articles arguing against antioxidant supplementation during training, highlighting that it could inhibit beneficial molecular, biochemical, and physiological adaptations [83].

In research conducted by Merry and colleagues, a considerable interindividual redox variability was observed, both at rest and in response to acute exercise. This heterogeneity was highlighted through the analysis of antioxidant biomarkers, such as glutathione and vitamin C, and oxidative stress markers, like F2-isoprostanes and carbonylated proteins [84]. This diversity among individuals provides a new perspective on the mixed results obtained in studies on the effectiveness of antioxidants as ergogenic aids. It was found that antioxidant treatments were usually administered to young and healthy individuals with normal levels of antioxidants or oxidative stress markers, which may explain why some participants did not experience significant benefits from these treatments [85].

Building on this finding, Michailidis adopted an innovative approach in biomedical research, known as stratified purposive sampling, to identify subgroups of individuals who might benefit most from antioxidant treatments. This strategy proposes a “stratified” approach to supplementation, personalizing the administration of antioxidants based on the specific redox profile of each individual [86]. Thus, the deficient antioxidant would be administered individually, contrary to the conventional practice of the unselective administration of antioxidants, whether it is a single type of antioxidant or a combination.

This “data-focused” methodology suggests that the impact of antioxidant supplements on adaptations and responses to exercise varies depending on the initial redox state of the individual. Therefore, the ergogenic benefits of antioxidant supplements become evident only in individuals with initially low levels of antioxidants or with high levels of oxidative stress [86]. This perspective not only balances the discussion around antioxidant supplements but also provides a clear direction for future research and for optimizing nutrition strategies in the context of physical exercise, highlighting the importance of personalizing nutritional interventions based on individual redox needs.

A relevant study in the military context explores the impact of training on oxidative stress and the role of antioxidant supplementation in this process. One specific example is research that investigates how antioxidant supplementation influences oxidative stress induced by physical training, concluding that although antioxidants can reduce oxidative stress, they may also interfere with some beneficial physiological adaptations to training, such as the enhancement of endogenous antioxidant capacity [87].

Antioxidants neutralize free radicals and protect against oxidative stress, reducing the potential for cellular damage and improving recovery. This is crucial in challenging environmental conditions where physical and chemical stress is intensified. A recent study examined oxidative stress markers and muscle damage in military cadets after an intensive 10-day training course followed by a one-month recovery period. The results showed an increase in myoglobin levels and a higher glutathione index, with significant improvements after the recovery period, highlighting a positive impact of the antioxidant system before and after training [88].

Antioxidant supplements are often used to mitigate negative responses to oxidative stress induced by intense training. However, it is important to note that while antioxidants can reduce oxidative stress biomarkers, they can also interfere with certain desired training adaptations, such as increased endogenous antioxidant capacity and mitochondrial biogenesis, as suggested by research published in *The Journal of Physiology* [84].

More research is necessary to produce evidence-based guidelines regarding the use of antioxidant supplements during training. The current recommendation is that an adequate intake of vitamins and minerals through a varied and balanced diet remains the best approach for maintaining optimal antioxidant status among physically active individuals [87].

The use of wearable devices and other advanced technologies allows for the continuous monitoring of oxidative stress biomarkers, facilitating the adjustment of antioxidant supplementation according to individual needs, and offering a personalized and dynamic approach to oxidative stress management [51].

Nutritional education is essential in promoting the correct and effective use of antioxidant supplements. Informing military personnel about the benefits and potential risks of antioxidant supplementation is crucial for adopting a balanced and well-informed approach. One study reveals that the use of antioxidant supplements is widespread among military personnel, highlighting the need to provide adequate education to ensure their safe and effective use [89].

A relevant example is the study conducted on military firefighters in Brazil, which investigated the metabolic response and certain oxidative stress markers in plasma and erythrocytes of firefighters supplemented or not with resveratrol (RES) for 90 days (100 mg/day). Analyses conducted before and after a typical physical fitness test used to induce oxidative stress showed that RES supplementation had no liver consequences compared to the placebo group. Although supplementation reduced levels of IL-6 and TNF-α after the fitness test, the effect on other oxidative stress biomarkers was not significant, suggesting that an antioxidant regimen might have an anti-inflammatory effect but not necessarily a major impact on antioxidant defense systems under conditions of moderate stress [90].

A study investigated the long-term effects of antioxidant supplementation, highlighting that antioxidants can influence the process of “intrinsic” aging as well as various pathological processes associated with aging. Long-term supplementation with vitamin E, for example, was associated with improvements in immune function in older subjects and the reduction of atherosclerosis risk, these benefits having direct implications for the long-term health of military personnel [91]. For a summary of the relevant studies and findings discussed in this chapter, see Table 4.

### 3.4. Antioxidant Supplements: Panacea, Harmful, or Neutral?

In recent decades, the role of reactive oxygen species produced during physical exercise has been profoundly re-evaluated. Initially seen as harmful by-products, these molecules are now recognized as essential signals that promote positive adaptations to exercise, such as mitochondrial biogenesis, angiogenesis, and neurogenesis, thereby contributing to the enhancement of physical performance. This new perspective highlights the complexity of the mechanisms through which exercise influences health and performance [92,93].

In this context, antioxidant supplements have traversed a controversial path, from being considered essential for combating the harmful effects of oxidative stress to being viewed as potentially inhibitory of the beneficial adaptations induced by exercise. The majority of relevant studies, including in vivo ones, have explored the use of antioxidant agents, reaching a consensus that supplementation either does not influence physical exercise adaptations or could even obstruct the beneficial effects of reactive species, resulting in a more reductive state than optimal and impeding adaptations to effort [94,95].

Similar observations regarding the neutral or even negative effects of antioxidant supplementation have been made in the context of the progression of diseases such as cancer and diabetes, as well as in increased mortality, contributing to the negative reputation of antioxidant supplements in the field of nutrition and biomedicine [96].

Research conducted by Sayin and colleagues revealed significant interindividual variability in redox responses both to antioxidant stress and to oxidative stress after acute exercise, in a sample of 100 participants. This heterogeneity was partially attributed to the baseline values of the measured biomarkers, suggesting that individual differences in redox state might play a key role in determining the effectiveness of antioxidants as ergogenic aids [97].

Furthermore, a study by Margaritelis and colleagues found that this redox individuality, assessed through exercise-induced changes in oxidative stress biomarkers such as F2-isoprostanes, could partially predict an individual’s aerobic and anaerobic trainability [98]. Based on this documented redox variability, it was proposed that baseline levels of antioxidants could also influence the physiology of physical exercise and nutritional outcomes. Therefore, Margaritelis and colleagues investigated in another study the impact of antioxidant supplementation, such as vitamin C and N-acetylcysteine (NAC), on exercise adaptations, finding that the ergogenic benefits of antioxidant supplements are evident only in individuals with specific deficiencies or with initially high levels of oxidative stress [99].

The exploration of the relationship between antioxidant supplements and oxidative stress in the context of physical exercise has evolved significantly, involving a reconsideration of the role of these supplements not only in the field of sports nutrition but also in general health. Recent research emphasizes that the effectiveness of antioxidant supplements in reducing oxidative stress and promoting health is significantly influenced by the individual’s initial redox state. This finding was exemplified by the study of Block and colleagues, which demonstrated that the benefits of supplementation with vitamin C or E on plasma levels of F2-isoprostane are limited exclusively to individuals with initially high levels of oxidative stress. Based on these results, the authors suggested a threshold of 50 μg F2-isoprostanes/mL in plasma as a benchmark for participant eligibility in studies targeting the use of antioxidants. This perspective underscores the importance of a more personalized approach in the research and application of antioxidant supplements, indicating that the response to antioxidant treatment can vary significantly depending on the person’s initial redox profile. Thus, to effectively assess the potential benefits of personalized nutrition, examining individual variability in responses to nutritional interventions is crucial. However, conducting such an assessment presents significant challenges, as precision nutrition, a pillar of personalized medicine, largely relies on identifying how an individual’s genetic characteristics influence the response to various nutritional interventions or supplements, such as nutrigenetics [100,101].

In this context, it is essential to consider factors such as dietary habits, physical activity level, and microbiome, which can significantly influence how an individual responds to nutritional intake [102]. In practice, nutritional advice based on detailed assessments of an individual’s diet or phenotypic markers, such as anthropometric measures and clinical variables, continues to form the foundation of personalized nutrition [103].

Studies have highlighted that specific deficiencies, such as low intake of vitamin C or sulfur-containing nutrients, could play a decisive role in an individual’s redox profiles, thus influencing the response to antioxidant supplementation. Identifying these deficiencies in groups with low antioxidant characteristics has enabled the application of targeted supplementation, which has effectively reversed these insufficiencies [104,105,106].

It is important to note that the current strategy focuses on applying personalized antioxidant treatments at the group level, based on the common identification of phenotypic or metabolic profiles, rather than on granular individual adjustments [107]. This approach, rooted in the concept of metabotyping, offers a promising path towards the broader implementation of personalized nutrition, in line with current trends in precision medicine. This evolution in understanding and applying antioxidant supplements reflects a move towards a more nuanced and individualized approach to nutrition, recognizing the complexity and diversity of human responses to nutritional interventions.

The development of understanding the role of antioxidant supplements in combating oxidative stress has been marked by significant evolution, especially in the context of physical exercise. The recognition that reactive oxygen species produced during physical activity are crucial for cellular signaling and inducing positive adaptations, such as mitochondrial biogenesis, angiogenesis, and neurogenesis, has shifted the paradigm regarding antioxidant supplements [92,93]. In this regard, it has been found that antioxidant supplementation can have varied effects, from neutral to even inhibitory of physiological adaptations to exercise, suggesting that their benefits or detriments largely depend on the individual’s initial redox state [94,95].

Recent explorations in personalized nutrition have highlighted that a more nuanced approach is needed to understand the impact of antioxidant supplementation. A specific clinical tool for identifying individual antioxidant deficiencies could optimize the effectiveness of antioxidant therapies, offering a promising route towards personalizing treatment based on each patient’s unique needs [100]. This perspective is supported by the discovery that the beneficial effects of supplements, such as vitamins C and E, are limited to individuals with initially high levels of oxidative stress, proposing a specific threshold of F2-isoprostane for identifying eligible candidates for such interventions [108].

Studies have drawn attention to significant interindividual redox variability in responses to exercise and supplementation, showing that groups with low levels of antioxidants exhibit initially inferior physical performance compared to those with moderate or high levels of antioxidants [107,109]. This observation underscores that the redox balance plays a crucial role in physical performance, and targeted supplementation can ameliorate specific deficiencies, significantly improving physical capacity and reducing oxidative stress.

It is essential to understand that antioxidant molecules do not act in isolation but are part of a complex defense system that includes both enzymatic and non-enzymatic mechanisms. Deficiencies in one of these components can affect the entire biochemical network, highlighting the importance of a holistic approach in assessing and treating oxidative stress [85]. Moreover, Paschalis and colleagues discovered that dysregulation in redox metabolism, such as in the GSH pathway, can lead to disruptions in the entire antioxidative machinery, with significant repercussions on systemic oxidative stress and physical performance [109].

The mechanisms through which the redox balance influences performance include regulating cellular signaling and energy metabolism through antioxidant enzymes, which act as key nodes in the redox network, selectively modulating signaling triggered by reactive species [85,110]. These enzymes exhibit high selectivity for signaling species and are kinetically favored over other antioxidants, highlighting the complexity and specificity of redox control over cellular functions [111,112,113,114,115].

Therefore, understanding how antioxidant enzymes and redox mechanisms regulate cellular signaling and energy metabolism opens the path to new strategies for enhancing physical performance and managing oxidative stress. This conceptual framework not only highlights the importance of redox balance in cellular health but also provides a basis for exploring personalized nutritional and supplemental interventions aimed at optimizing health and performance.

In the context of oxidative stress and the response to physical exercise, understanding the mechanisms through which energy metabolism is regulated becomes essential. One of the most relevant signaling pathways involved in this process is the regulation of glucose uptake, particularly through the glucose transporter 4 (GLUT4). This pathway is especially important during physical exertion when the body’s energy demand significantly increases [116].

Reactive oxygen species [ROS] and reactive nitrogen species (RNS), including nitric oxide (NO), play a crucial role in regulating this process. An increasing body of evidence underscores the influence of NO on muscle contraction-stimulated glucose uptake. Similarly, in vitro and ex vivo studies indicate a similar effect of ROS on glucose uptake, though this has not yet been confirmed under in vivo conditions [117,118,119]. This highlights the complex and nuanced role of reactive species in the metabolic regulation of muscle cells during physical activity.

Besides these signaling mechanisms, it is well established that the functionality of many enzymes is directly influenced by their oxidation state. This is particularly relevant for enzymes involved in energy metabolism, such as creatine kinase, which plays an essential role in maintaining and recycling ATP in muscle cells [120]. A disturbed redox state can, therefore, have significant implications for energy production capacity during physical exercises, underscoring the importance of redox balance for optimal energy metabolism functioning.

The importance of finely regulating these signaling pathways and energy metabolism through the antioxidant defense system becomes evident. Antioxidants, especially antioxidative enzymes, play a vital role in maintaining this balance, counteracting the potentially harmful effects of an imbalanced redox state [121]. This balance is crucial not only for preventing oxidative damage but also for ensuring adequate cellular signaling and energy metabolism, especially under the increased physical stress encountered during physical exercises.

A study on antioxidant protection against cosmic radiation at commercial flight altitudes suggests that cosmic radiation can affect the human body and induce oxidative stress. This highlights the importance of prophylactic antioxidant treatment for individuals exposed to such conditions, including military personnel deployed in areas with intense solar exposure or at high altitudes [122].

Research on volunteers from the Marine Firefighters Corps has shown that antioxidant supplementation can significantly reduce oxidative stress markers at moderate altitudes, although the effects varied depending on the specific biomarkers assessed. This underscores the need for well-tuned supplementation strategies to maximize benefits and minimize any potential negative effects [123].

There is evidence that antioxidant supplementation, such as with vitamins C and E, might inhibit certain beneficial physiological adaptations to endurance training, such as mitochondrial biogenesis, essential for enhancing endurance performance and overall health. For instance, a study demonstrated that supplementation with vitamins C and E attenuated the increase in mitochondrial proteins induced by endurance training, suggesting that supplements might interfere with the cellular signals necessary for beneficial exercise adaptations [124].

Another study investigated the effects of combining vitamin C and E supplementation on various measures of exercise performance after endurance training. The results suggest that administering vitamins C and E to healthy individuals without prior vitamin deficiencies had no effect on physical adaptations to intense endurance training, indicating that supplements did not enhance physical performance and might even limit beneficial adaptations [125].

Education on antioxidants should cover both their benefits in protecting against oxidative stress and the potential risks of inhibiting physiological adaptations to intense physical exercise. Integrating case studies and the latest research into courses can provide military personnel with a deeper understanding of the complexity of antioxidant effects [87].

Initiating longitudinal studies to track the long-term effects of antioxidant use on military health and performance is essential to determine the real benefits and risks associated with antioxidant supplements. A notable example of a longitudinal study that assessed the impact of daily supplementation with antioxidant vitamins and minerals is the SU.VI.MAX (Supplémentation en Vitamines et Minéraux AntioXydants) Study. Participants received a daily mix of vitamin C, beta-carotene, vitamin E, selenium, and zinc, or a placebo, over a period of 8 years. The results indicated potential benefits of antioxidant supplementation on cognitive performance but require further evaluation in the context of military stress and long-term physical performance [126]. For a summary of the relevant studies and findings discussed in this chapter, see Table 5.

### 3.5. Nutritional Supplements: Synergies and Divergences with Antioxidants

In discussing the efficacy of antioxidant supplementation, an emerging viewpoint suggests that the benefits of these supplements might be best realized by individuals who exhibit specific deficiencies or have low baseline levels of antioxidants. This perspective extends not only to antioxidants but also to other nutritional supplements, including vitamins E and D, recognized for their capacity to counteract molecular and biochemical disorders and to alleviate adverse physiological conditions [127].

Vitamin E, particularly known in the literature as α-tocopherol, is valued for its lipophilic antioxidant properties and its role in regulating cellular metabolism. However, studies indicate that a significant percentage of the adult population does not achieve the recommended intake of vitamin E through diet, with estimates showing that between 80 and 90% of adults consume insufficient quantities of this vitamin [128]. This nutritional deficit is associated with a variety of negative symptoms, from anemia and increased vulnerability to infections, to cognitive dysfunctions and developmental problems [129].

Mechanistic research, mostly conducted on animal models such as rats and zebrafish, has discovered that long-term deficiencies in vitamin E can lead to a range of complications, including the dysregulation of energy metabolism, neurological dysfunctions, suboptimal lipid profiles of tissues, compromised mitochondrial function, and extensive tissue damage caused by the peroxidation of polyunsaturated fatty acids (PUFAs) in membranes [130,131]. Additionally, it has been observed that long-lasting disruptions in redox homeostasis and cellular metabolism induced by the lack of vitamin E can persist even after dietary or supplemental correction, and in extreme cases, severe deficiency has been linked to embryonic mortality [132,133].

Optimistically, supplementation with higher doses of vitamin E can significantly increase its concentrations in the body, succeeding in reversing multiple comorbidities and providing tangible health benefits [131]. However, the effects of the long-term consumption of vitamin E in doses far exceeding optimal levels remain a subject of controversy [134,135]. This underscores the need to carefully balance the intake of vitamins and antioxidant supplements to support cellular and metabolic health without risking the potential negative effects of nutritional excess. Thus, understanding and correctly applying nutritional supplementation requires a personalized approach that considers the specific nutritional state and individual needs of each person, highlighting the importance of careful assessment and appropriate clinical monitoring.

Exploring the role of vitamins D and E in the context of health and physical performance has revealed that deficiencies in these essential nutrients are surprisingly common, both among athletes and in the general population. This underscores the importance of a careful approach to dietary supplementation, considering that deficiencies can have serious consequences on muscle function, immunity, bone health, and cardiovascular function. In particular, vitamin D has garnered attention in recent years due to its association with a wide range of comorbidities related to its deficit. Studies have shown that maintaining optimal levels of vitamin D is crucial for muscle regeneration and can improve recovery capacity and hypertrophic response after eccentric exercises [136,137,138,139].

Interestingly, similar to the approach to antioxidants, the efficacy of supplementation with vitamins D and E is seen to be greatest in individuals with initially low levels of these nutrients. Despite the vital role of vitamin E as a lipophilic antioxidant and regulator of cellular metabolism, a large number of adults do not meet the dietary requirements for this vitamin, leading to various negative symptoms [127,128]. Studies on animal models have indicated that long-term deficiencies in vitamin E can lead to serious issues, including the dysregulation of energy metabolism and disturbed mitochondrial function, highlighting the necessity of careful supplementation to counter these effects [130,131].

However, it is crucial to note that chronic supplementation with high doses of vitamins D and E does not always yield additional benefits and, in some cases, may even be counterproductive [140]. This emphasizes a need for balance and personalization in the use of supplements, indicating that personalized nutritional interventions, which consider the individual’s specific deficiencies, can provide significant improvements in physical performance and the redox profile [108,109].

This perspective is reinforced by the observation that a stratified nutritional approach, focusing on the individual phenotype rather than the genotype, can offer a promising pathway to optimizing health and performance. This involves using personalized antioxidant supplementation strategies and other essential nutrients to ensure optimal levels during sports competitions or to support recovery in clinical contexts [141].

In conclusion, the attempt to refine the approach suggested by Halliwell, which proposes testing antioxidants on individuals “more susceptible” to disease risk, urges us to identify and address specific antioxidant deficiencies for each individual. This underscores the potential of personalized nutrition, based on identifying individual nutritional needs and applying targeted interventions to maximize health and performance benefits [142].

Tailoring nutritional supplements, including antioxidants, to meet the specific needs of military personnel is crucial as they often face intense physical and psychological stress. Personalizing supplementation can optimize performance and resilience under varying mission conditions. Nutritional supplements, including antioxidants, can be adapted to support specific needs of military personnel such as enhancing resistance to physical stress and rapid recovery from injuries. For example, supplementation with magnesium and vitamin B12 for protection against loud noises, glutamine and omega-3 for improving trauma recovery, beta-alanine for intense physical activity, and caffeine for enhancing mental function are all beneficial [87].

Recent studies indicate that antioxidants, such as vitamins C and E, can help maintain physical performance even under conditions of intense oxidative stress, typical of pro-longed physical exercises or military operations. For instance, a study demonstrated that antioxidant supplementation could reduce oxidative stress induced by intense exercise, although the impact on physical performance is still a subject of debate [143].

Vitamin E, due to its strong antioxidant properties, plays a crucial role in protecting against UV-induced injuries, which can accelerate oxidative stress and contribute to skin and other exposed tissues’ deterioration. Supplementation with vitamin E has been associated with a reduction in the harmful effects of UV radiation, reducing the potential for skin cancer and other dermatological conditions exacerbated by sun exposure.

Regular nutritional assessments can identify specific needs of soldiers, such as vitamin and mineral deficiencies or increased antioxidant needs due to heightened oxidative stress in military exercises. For example, a study demonstrated the importance of regular assessments to tailor supplementation to operational requirements and the specific stress of each mission [144].

Education on the importance and correct use of nutritional supplements is crucial for military personnel. Training modules about supplements, including when and how to use them effectively, can prevent misuse and maximize the benefits of supplementation. A relevant example is the development of a web-based educational module for military healthcare providers, teaching them how to assess and communicate the evidence-based literature about supplements [145].

Studies must evaluate the safety of supplements, ensuring there are no long-term adverse effects and that supplements do not interfere with natural physiological adaptations to stress and training. A study assessed the effect of antioxidants in diet and supplementation, pharmaco-nutritional support, and the use of an anti-inflammatory in a group of patients with advanced cancer and associated anorexia–cachexia. The results indicated a significant improvement in nutritional status and quality of life, suggesting that an integrated treatment including antioxidants can be effective and safe [146].

Integrating training modules into the curriculum of military academies and continuing education programs. These modules should cover topics such as the mechanisms of action of supplements, proper dosing, and management of side effects.

It’s important to develop and distribute educational materials, like brochures and online tutorials, that offer accessible, evidence-based information on supplement use.

Another important aspect is conducting periodic assessments of nutrition and supplement knowledge among military personnel to keep the information relevant and current. For a summary of the relevant studies and findings discussed in this chapter, see Table 6.

## 4. Discussion

### 4.1. The Impact of Physical Exercise on Oxidative Stress in Military Missions

*Importance of the Subject*

Regular physical exercise is essential for maintaining and enhancing the resilience and performance of military personnel, playing a crucial role under conditions of extreme stress. These activities not only improve psychological and physical resilience but are fundamental in the efficient management of oxidative stress, a vital element for accomplishing critical missions.

*Analysis and Observations*

Physical exercises significantly contribute to strengthening endurance and strength, necessary for meeting the physical requirements in combat scenarios or extended missions. These are essential for supporting mental health, helping to reduce the impact of psychological stress in conflict situations [44]. Additionally, regular training under extreme conditions can stimulate the increase of antioxidant capacity, protecting against cellular damage induced by free radicals [143]. Furthermore, physical exercise promotes mitochondrial adaptations, enhancing efficiency in energy production and reducing the production of reactive oxygen species, crucial for maintaining performance under severe stress [147].

*Conclusions*

Integrating technology into training regimes can amplify the benefits of physical exercise. Wearable devices and other modern technologies offer advanced methods for monitoring and enhancing physical performance and managing oxidative stress, essential for tailoring the training to individual needs [51].

*Recommendations*

For optimizing training, we recommend implementing real-time feedback and using advanced biochemical analysis to monitor oxidative stress markers such as malondialdehyde (MDA) or superoxide dismutase (SOD), allowing for precise evaluations and adjustments of antioxidant strategies post-training [52]. These measures help guide the intensity and duration of the training to ensure that each session is within the optimal limit for enhancing performance and minimizing stress.

*Implementation Recommendations*

It is recommended to use the data collected through advanced technologies for the personalized adjustment of training, optimizing the balance between oxidative stress and recovery. It is crucial to monitor progress and adjust training plans to reflect improvements in physical performance and oxidative stress management. Therefore, integrating these strategies into military training not only makes them more effective but also safer, reducing the risk of injury and enhancing the overall health and performance of military personnel. This data-driven approach represents a significant evolution in military training, offering unprecedented opportunities for enhancing adaptability and responsiveness to individual needs.

### 4.2. The Influence of the Ketogenic Diet in Optimizing Performance and Regulating Oxidative Stress in Military Missions

*Importance of the Subject*

Traditionally, military nutrition has focused on caloric intake and nutrients to support physical exertion, but recently, the ketogenic diet (KD) has been recognized for its potential to improve the management of oxidative stress and to enhance the resilience of military personnel in extreme stress conditions. This diet is defined by low carbohydrate intake and increased fat consumption, supported by research indicating positive effects on mitochondrial function and a general reduction in reactive oxygen species, thus helping to reduce oxidative stress [56,57].

*Analysis and Observations*

Studies have shown that KD positively impacts the efficient management of energy and body composition, associated with significant weight loss and reductions in visceral fat stores, without negatively affecting physiological adaptations to intense physical training [80]. This regimen provides a steady and efficient energy source, which is vital in military mission scenarios where traditional food options are limited [81].

*Conclusions*

In addition to supporting physical and mental well-being, KD facilitates the shift from glucose to ketone bodies as the primary energy source. This metabolic change not only prevents sudden energy fluctuations but also enhances endurance and capacity for prolonged effort [148]. The importance of diets in reducing systemic inflammation and accelerating recovery after exercise is well documented, with KD helping to minimize muscle soreness and shorten recovery times [79]. Furthermore, the antioxidative effects of the diet protect cells and the brain, improving cognitive functions vital for soldier performance [149].

*Recommendations*

The adoption of KD should be preceded by rigorous and personalized evaluations to determine the most effective adaptation to the individual requirements of each soldier. This includes initiating pilot programs and a system of continuous monitoring of the diet’s impact on performance and military health [80].

*Implementation Recommendations*

It is vital that the adoption of KD is accompanied by careful supervision and detailed research to validate the long-term impact of the diet on military performance and health. Its implementation needs to be flexible, able to adapt to various environmental conditions, and effectively respond to different levels of stress [78,79]. Additionally, it is essential to develop nutritional protocols that adhere to ethical standards and are sustainable, to ensure that the dietary strategy can be integrated long-term into the practices of the armed forces. These measures will facilitate the successful integration of KD into military diets, maximizing benefits and minimizing associated risks.

### 4.3. Efficacy and Limitations of Antioxidants in a Military Context

*Importance of Antioxidants*

Antioxidants are essential in neutralizing the oxidative stress that occurs in extreme conditions, such as those encountered in military, naval, and space environments. The introduction of antioxidant supplements is crucial for maintaining redox balance and protecting against cellular damage caused by free radicals, making it a vital component in ensuring the health of personnel under these conditions [51].

*Extreme Conditions and Antioxidant Response*

Exposure to extreme conditions, such as high temperatures or intense noise and vibrations, can increase the production of free radicals. The use of antioxidants helps stabilize the redox balance by neutralizing these radicals, protecting the body against oxidative stress, and facilitating a quick and effective recovery [87].

*Impact of Antioxidants on Military Performance*

Antioxidant supplementation contributes to maintaining physical performance and enhancing the endurance and recovery capabilities of soldiers, which are essential for the success of long-duration military operations [88].

*Advanced Technology in Monitoring Oxidative Stress*

The use of cutting-edge technology, including wearable devices, allows for the detailed monitoring of oxidative stress biomarkers. This facilitates a personalized and dynamic approach in adjusting antioxidant supplementation, tailored to the individual needs of each military personnel [51].

*Limitations and Considerations of Supplementation*

Research indicates that while antioxidants reduce oxidative stress, they can interfere with beneficial adaptations to training, such as the enhancement of endogenous antioxidant capacity and mitochondrial biogenesis. Therefore, it is crucial to consider these aspects when formulating supplementation programs [84,87].

*Conclusions and Recommendations for Implementation*

It is essential to carefully evaluate both the benefits and potential adverse effects of antioxidant supplements in the military context. A personalized and rigorously monitored supplementation strategy is recommended, which includes regular assessments of the redox state and oxidative stress, to customize and adjust supplementation according to specific needs [51]. The ongoing education of military personnel about the benefits and risks associated with antioxidant supplements is also essential. Further studies in military environments are necessary to validate the effectiveness and safety of these supplements, adjusting programs accordingly to enhance the recovery capacity and performance of military personnel, contributing to the creation of a more resilient and prepared workforce. These strategic measures can fundamentally transform the health and operational capacity of the armed forces, ensuring a healthier and more efficient workforce.

### 4.4. Antioxidant Supplements: A Critical Analysis of the Benefits and Risks for Military Personnel

*Role and Benefits of Antioxidants*

Antioxidant supplementation is widely recognized for its ability to counteract oxidative stress, a significant factor in cellular damage under extreme conditions, such as those encountered in military missions. Studies highlight the protective role of antioxidants against cellular damage in scenarios such as exposure to intense solar radiation or at high altitudes, where oxidative stress is intensified [122].

*Controversies and Limitations of Supplementation*

Although antioxidants are valuable in managing oxidative stress, studies indicate they can interfere with beneficial physiological adaptations to intense training. Supplementation with vitamins C and E, for example, can attenuate the increase in the production of new mitochondria in muscles, a crucial aspect for adaptations to endurance training [124,125].

*Assessments and Recommendations*

It is essential to conduct critical assessments of the benefits and disadvantages of antioxidants before integrating them into military diets. Longitudinal studies, such as the SU.VI.MAX (Supplémentation en Vitamines et Minéraux AntioXydants), provide valuable insights into the long-term effects of supplements and can guide the evidence-based use of antioxidants in the armed forces [126].

*Implications and Monitoring*

Antioxidants can play a dual role, offering protection in extreme conditions, but also potentially inhibiting the natural adaptations necessary for intense physical exercise. Therefore, their use must be balanced and well monitored to ensure that the benefits outweigh the associated risks.

*Implementation Strategies*

The effective implementation of antioxidant supplements requires a prudent and well-documented approach. Personalized monitoring and ongoing education are essential to ensure that military personnel maintain long-term performance and health. It is crucial to develop supplementation protocols that are tailored to the specific needs and conditions of each soldier, thus ensuring a workforce well prepared for the challenges of military service.

### 4.5. Interactions and Implications of Nutritional Supplements in Combating Military Oxidative Stress

*Importance of Personalized Supplementation*

Tailoring nutritional supplements to the specific requirements of military personnel is crucial for enhancing their resistance to physical stress and for rapid recovery after injuries. Adjusting supplements such as magnesium, vitamin B12, glutamine, omega-3, beta-alanine, and caffeine proves essential, each playing specific roles in protecting against intense noise and in improving recovery after trauma and intense physical activity [150].

*Periodic Assessments and Nutritional Education*

Periodic assessments of antioxidant status and other health markers are vital to personalize nutritional supplementation. These evaluations ensure that each soldier receives the optimal dose to support health without compromising training adaptations. Also, ongoing education on the efficient and safe use of antioxidant supplements is crucial, emphasizing the need for a balanced approach.

*Conclusions and Recommendations*

It is imperative to develop and implement personalized supplementation protocols, tailored to the varied environmental conditions and stress, to ensure that nutritional supplements, including antioxidants, are used effectively. These protocols should be flexible and well adapted to the specific requirements of military missions, ensuring efficient integration into the daily regimes of the personnel.

*Benefits of Antioxidants in Military Context*

Antioxidants are essential for maintaining cellular integrity and operational performance, playing a crucial role in preventing muscle fatigue and accelerating recovery after intense physical training. They help neutralize free radicals that accumulate under conditions of extreme physical stress, thereby reducing the risk of cellular damage and enhancing cognitive function in challenging conditions [143].

*Recommendations for Implementation*

Implementing regular personalized assessments is recommended to maximize the benefits of antioxidants without compromising physiological adaptations to training. Additionally, it is crucial to provide ongoing education in military academies to inform soldiers about the benefits and risks of using antioxidants. This should include discussions on the importance of a balanced diet rich in natural antioxidants and the conditions under which supplementation might be beneficial. These strategies ensure that military personnel are well prepared and capable of maintaining optimal performance under pressure, minimizing the risks associated with intense oxidative stress conditions.

## 5. Conclusions

This narrative review has underscored the paramount importance of physical exercise and dietary adaptations, including the benefits of the ketogenic diet, in enhancing the resilience and performance of military personnel against oxidative stress. Emphasizing the role of physical activity in improving cardiovascular, cognitive, and musculoskeletal health, the study underscores how an active lifestyle can counteract the negative impact of oxidative stress, which is essential for the successful completion of critical missions.

The integration of modern technologies, such as wearable devices and smart sensors, into military training allows for the detailed monitoring and real-time adjustment of exercise regimes, maximizing the efficiency and safety of training under extreme conditions. These technological adaptations provide unprecedented opportunities for enhancing physical performance and effective management of oxidative stress.

The diversity of individual responses to oxidative stress highlights the need for personalized intervention strategies, tailored based on the unique redox profile and specific requirements of each staff member. We recommend the development and implementation of personalized and well-monitored supplementation protocols, including periodic assessments and ongoing education to optimize the use of antioxidants and other nutritional supplements.

Furthermore, the ketogenic diet, characterized by low carbohydrate intake and high fat content, has shown potential in improving mitochondrial function and reducing the production of reactive oxygen species, thus offering an efficient solution for managing oxidative stress under extreme stress conditions, typical of prolonged military missions.

This synthesis makes a valuable contribution to the specialized literature, proposing a comprehensive framework for intervention and initiating new directions for future investigations. The results of this analysis provide the foundation for the development of more effective and personalized intervention strategies, essential for enhancing performance and maintaining health in the context of the inherent challenges of military activities.

## Figures and Tables

**Table 1:** Research Questions.

Nr.	Question of Research	Description
1	Interindividual variability and exercise response	Investigates how individual differences affect the effectiveness of physical exercise in attenuating oxidative stress, highlighting the need for personalized training regimens.
2	Characteristics of the optimal diet	Explores the features of a diet that maximizes adaptation to extreme oxidative stress, emphasizing the identification of specific diets that offer maximum protection.
3	Synergy between exercise and nutrition	Analyzes how regular physical exercise and a personalized diet work together to combat oxidative stress, with a focus on the underlying biological mechanisms.
4	Development of personalized strategies	Proposes the design and implementation of personalized intervention strategies to efficiently respond to the diversity of military personnel needs, recognizing the complexity of individual experiences.

**Table 2:** Key Findings and Contribution to Physical Exercise.

Author(s) and Year	Key Findings and Contribution to Physical Exercise
Hussain et al. (2022) [13]	Discussed the broad health benefits of physical exercise (EXR), emphasizing its role in improving cardiovascular health and cognitive function, and reducing chronic disease risks.
Lisi et al. (2023) [14]	Explored how physical exercise promotes adaptations in extracellular vesicles that help manage oxidative stress, highlighting a novel pathway for its beneficial effects.
Fragala et al. (2019) [15]	Outlined the importance of resistance training in enhancing cardiopulmonary fitness and overall health, especially in older adults.
Jang et al. (2020) [16]	Validated exercise equations for assessing cardiorespiratory fitness, demonstrating their practical utility in clinical settings.
Helgerud et al. (2022) [17]	Demonstrated how submaximal exercises can predict VO2max, crucial for evaluating cardiovascular improvements due to EXR.
Schroeder et al. (2019) [18]	Highlighted the cardiovascular benefits of combining different training modalities, contributing to reduced disease risk factors.
Haverkamp et al. (2020) [19]	Showed significant cognitive improvements from physical activity, establishing a link between aerobic capacity and cognitive health.
Lake et al. (2022) [20]	Discussed the cerebrovascular responses to aerobic training in older adults, emphasizing its role in enhancing brain health and function.
Caplin et al. (2021) [21]	Investigated the effects of exercise intensity on stress responses, indicating protective benefits against stress via cortisol regulation.
Ryan et al. (2020) [22]	Demonstrated similar benefits of moderate and high-intensity training on insulin sensitivity, relevant for metabolic health.
Bobinski et al. (2018) [23]	Discussed the analgesic effects of low-intensity exercise in neuropathic pain management, mediated by interleukin-4.
Otsuka et al. (2022) [24]	Reported positive effects of resistance training intensity on muscle quality in older individuals, important for maintaining physical function.
Sailani et al. (2019) [25]	Linked lifelong physical activity to beneficial genetic expressions related to metabolism and oxidative stress resistance in aged muscle.
Hargreaves and Spriet (2020) [26]	Examined how exercise influences skeletal muscle energy metabolism, crucial for enhancing endurance and performance.
Sorriento et al. (2021) [27]	Highlighted the protective role of physical exercise on mitochondrial health, a key factor in cellular energy management and longevity.
Steinbacher and Eckl (2015) [28]	Discussed the impact of oxidative stress on exercising muscle, underlining the dual role of exercise in managing oxidative balance.
Powers et al. (2020) [29]	Analyzed exercise-induced oxidative stress as both a beneficial and challenging aspect of regular physical activity.
Henríquez-Olguin et al. (2019) [30]	Showed how exercise-induced ROS influences glucose uptake during physical activity, linking to metabolic health improvements.
Matta et al. (2022) [31]	Investigated the effects of exercise on white adipose tissue, showing improvements in redox homeostasis and mitochondrial function.
Baird and Yamamoto (2020) [32]	Explored the molecular mechanisms of the KEAP1-NRF2 pathway in oxidative stress regulation, pivotal for cellular defense during exercise.
Papanikolaou et al. (2023) [33]	Connected dietary intake, physical fitness, and redox status, illustrating the holistic impact of exercise and nutrition on health.
Pinto et al. (2022) [34]	Investigated gender differences in response to exercise training, important for tailored physical education and training programs.
Moreira-Reis et al. (2022) [35]	Demonstrated improvements in cardiovascular and muscular fitness through aerobic dance, emphasizing its benefits for the elderly.
Gonzalez-Gil and Elizondo-Montemayor (2020) [36]	Reviewed the complex interactions between different body-produced factors during exercise, enhancing understanding of its systemic benefits.
Chow et al. (2022) [37]	Highlighted the role of exerkines in mediating the health benefits of exercise, expanding the understanding of inter-organ communication.
Thyfault and Bergouignan (2020) [38]	Discussed the broader metabolic health impacts of exercise, beyond just muscle adaptation, showing its influence on overall metabolic health.
Yáñez-Mó et al. (2015) [39]	Described the physiological functions of extracellular vesicles, which play significant roles in cellular communication during exercise.
Gruenberg (2020) [40]	Explored the role of multivesicular bodies in cellular processes, relevant to understanding how exercise affects cell biology.
Whitham et al. (2018) [41]	Elaborated on how extracellular vesicles mediate tissue crosstalk during exercise, facilitating the systemic health benefits of physical activity.
Alberro et al. (2021) [42]	Discussed the sources and effects of extracellular vesicles in blood, highlighting their applications in health and disease contexts influenced by exercise.
Hink et al. (2001) [43]	Examined the mechanisms of endothelial dysfunction in diabetes, showing how exercise can positively influence vascular health.
Szivak and Kraemer (2015) [44] **	Emphasized the role of physical exercise in enhancing physiological readiness and resilience, crucial for military preparedness.
Chang et al. (2023) [45] **	Demonstrated that physical fitness programs significantly reduce risk factors for metabolic syndrome among military personnel, underscoring the health benefits of regular exercise in the military.
Hedlund et al. (2015) [46] **	Showed that structured workouts improve communication and cooperation among military units, enhancing team cohesion and effectiveness in operations.
Manning and Fullerton (1988) [47] **	Found that high cohesion in military units, facilitated through joint physical training, leads to improved well-being and satisfaction in military careers.
Adler et al. (2015) [48] **	Reported that resilience training during Basic Combat Training reduces psychological stress and enhances stress management, promoting positive morale among soldiers.
Kyröläinen et al. (2018) [49] **	Highlighted the importance of integrating endurance and strength training to optimize physical performance in military environments.
Vantarakis et al. (2017) [50] **	Found that linear periodized resistance training significantly improves musculoskeletal fitness and specific conditioning needs of naval cadets, emphasizing tailored training benefits.
Jovanov et al. (2003) [51] **	Discussed the use of wireless sensor systems for continuous monitoring of physiological responses during intense military training, aiding in personalized training adjustments.
Tanskanen et al. (2011) [52] **	Studied the association of military training with oxidative stress, highlighting the need for monitoring and managing oxidative stress in training programs.
Hall et al. (2020) [53] **	Demonstrated that physical exercise significantly enhances physical function and reduces clinical risk factors for chronic diseases in older military veterans with PTSD.

** Military context.

**Table 3:** Key Findings and Contribution to Ketogenic Diet and Oxidative Stress.

Author(s) and Year	Key Findings and Contribution to Ketogenic Diet and Oxidative Stress
Allen et al. (2013) [54]	Demonstrated that the ketogenic diet enhances oxidative stress responses and therapy outcomes in lung cancer, underscoring its potential for improving mitochondrial metabolism.
Atakan et al. (2021) [55]	Identified high-intensity interval training’s enhancement of exercise capacity and health, illustrating how such exercise, potentially alongside ketogenic diets, optimizes mitochondrial metabolism and reduces oxidative stress.
Pinto et al. (2018) [56]	Highlighted the antioxidant and anti-inflammatory activities of the ketogenic diet, showing its neuroprotective potential in Alzheimer’s disease through reducing oxidative stress.
Reuter et al. (2010) [57]	Explored the links between oxidative stress, inflammation, and cancer, suggesting that the ketogenic diet could mitigate these pathways by efficiently managing oxidative stress.
Kodydkova et al. (2013) [58]	Investigated oxidative stress markers in chronic conditions, reinforcing how ketogenic diet-induced adjustments in oxidative balance could protect against cellular damage.
Lin and Karin (2007) [59]	Discussed cytokine-mediated links between inflammation and cancer, relevant to how ketogenic diets might modulate inflammation and oxidative stress in clinical settings.
Ostan et al. (2015) [60]	Reviewed the impact of diet on inflammaging and cancer, emphasizing the role of ketogenic diets in reducing inflammation-driven oxidative stress.
Susan (2005) [61]	Examined how ketogenic diets modulate oxidative stress and mitochondrial function, providing a protective mechanism against tumor development and oxidative damage.
Poff et al. (2013) [62]	Showed that the ketogenic diet and hyperbaric oxygen therapy prolong survival in mice with systemic metastatic cancer, highlighting the role of ketosis in oxidative stress management.
Nazarewicz et al. (2007) [63]	Analyzed the redox status of human blood on a short-term ketogenic diet, noting improvements in antioxidant capacity without inducing oxidative stress.
Sullivan et al. (2004) [64]	Demonstrated that the ketogenic diet increases levels and activity of mitochondrial uncoupling proteins, potentially reducing ROS and enhancing mitochondrial health.
Tieu et al. (2003) [65]	Discussed the neuroprotective effects of β-Hydroxybutyrate, a ketone body, showing its role in enhancing mitochondrial respiration and reducing oxidative stress through the NRF2 pathway.
Chorley et al. (2012) [66]	Identified novel NRF2-regulated genes, indicating how the ketogenic diet might influence antioxidant pathways and detoxification.
Shimazu et al. (2013) [67]	Explored the role of β-Hydroxybutyrate in suppressing oxidative stress and acting as a histone deacetylase inhibitor, facilitating protective gene transcription.
Yang et al. (2016) [68]	Highlighted how the ketogenic diet protects mitochondrial DNA against oxidative damage, supporting mitochondrial health in an animal study.
Jarrett et al. (2008) [69]	Found that the ketogenic diet increases mitochondrial glutathione levels, suggesting its role in enhancing the body’s internal antioxidant system and protecting cells from oxidative stress.
Elamin et al. (2018) [70]	Demonstrated that the ketogenic diet modulates NAD+-dependent enzymes and reduces DNA damage in the hippocampus, indicating its potential protective effects against oxidative and metabolic damage in malignant brain tissue.
Stafford et al. (2010) [71]	Showed that the ketogenic diet reverses gene expression patterns and reduces levels of reactive oxygen species in glioma, highlighting its ability to modulate oxidative stress beneficially.
Alhamzah et al. (2023) [72]	Reviewed the effects of the ketogenic diet on oxidative stress and cancer, underscoring the need for further research to confirm its anticancer benefits due to the current limitations in controlled studies.
Xu et al. (2020) [73]	Explored the direct association between diet and oxidative stress development, pointing out that dietary factors significantly influence oxidative stress, a common pathogenic mechanism in chronic diseases.
Kong et al. (2022) [74]	Investigated the link between dietary diversity and oxidative stress in older adults, finding that a higher-quality diet is associated with improved Total Antioxidant Capacity (T-AOC), which indicates a better balance of oxidative stress.
Aleksandrova et al. (2021) [75]	Conducted a systematic review of dietary patterns and their impacts on biomarkers of oxidative stress and inflammation, reinforcing the connection between diet diversity and nutritional status in managing oxidative stress.
Zhang et al. (2017) [76]	Highlighted the beneficial effects of cherry juice on inflammation and oxidative stress biomarkers, supporting the diet’s capacity to modulate these processes effectively.
Newman et al. (2017) [78]	Found that the ketogenic diet reduces mid-life mortality and enhances memory performance in aging mice through mechanisms that include decreased insulin levels and improved mitochondrial efficiency.
Greco et al. (2016) [79]	Showed that the ketogenic diet improves mitochondrial function and reduces oxidative stress, contributing to better energy efficiency and the maintenance of muscular integrity under prolonged physical stress.
LaFountain et al. (2019) [80] **	Observed significant health improvements among military personnel on a ketogenic diet, noting reductions in weight, body fat, and enhanced physical performance necessary for training, suggesting overall health and readiness benefits.
Volek et al. (2019) [81] **	Discussed how a ketogenic diet supports the maintenance of physical and cognitive function in challenging conditions, providing a stable and efficient energy source derived from ketone bodies, valuable in scenarios where food access is limited.

** Military context.

**Table 4:** Key Findings and Contribution to Antioxidants.

Author(s) and Year	Key Findings and Contribution to Antioxidants
Tan and Norhaizan (2019) [82]	Initially viewed antioxidants as crucial for combating exercise-induced oxidative stress due to their role in managing reactive oxygen species.
Nikolaidis et al. (2012) [83]	Highlighted a shift in perspective, suggesting antioxidant supplements could inhibit beneficial adaptations to exercise, such as molecular and physiological improvements.
Merry and Ristow (2016) [84]	Observed considerable interindividual variability in redox responses to exercise, influencing the effectiveness of antioxidants as ergogenic aids.
Paschalis et al. (2018) [85]	Found that antioxidant benefits, such as improved exercise performance and reduced oxidative stress, were primarily evident in individuals with initially low antioxidant levels.
Michailidis et al. (2013) [86]	Proposed a stratified approach to antioxidant supplementation, personalizing it based on individual redox profiles to optimize benefits.
Peternelj and Coombes (2011) [87] **	Discussed the dual role of antioxidants in potentially reducing oxidative stress while possibly interfering with training adaptations like endogenous antioxidant capacity enhancement.
Plavina et al. (2021) [88] **	Demonstrated that antioxidants help manage oxidative stress and aid recovery post-intensive training, emphasizing the importance of recovery periods.
Merry and Ristow (2016) [84] **	Reinforced the notion that while antioxidants can reduce oxidative stress markers, they might also impede desired adaptations such as mitochondrial biogenesis.
Knapik et al. (2018) [89] **	Reported widespread use of antioxidant supplements among military personnel, stressing the need for proper education on their benefits and risks.
Macedo et al. (2015) [90] **	Investigated the effects of resveratrol on military firefighters, noting anti-inflammatory benefits but limited impact on other oxidative stress biomarkers.
Meydani et al. (1998) [91]	Explored long-term antioxidant supplementation, finding benefits such as improved immune function and reduced atherosclerosis risk, relevant to military personnel’s long-term health.

** Military context.

**Table 5:** Key Findings and Contributions on the Role of Antioxidants: Beneficial, Harmful, or Neutral.

Author(s) and Year	Key Findings and Contributions on the Role of Antioxidants: Beneficial, Harmful, or Neutral
Margaritelis et al. (2018) [92]	Highlighted the beneficial role of reactive oxygen species in promoting adaptations like mitochondrial biogenesis and neurogenesis, thereby enhancing physical performance.
Mason et al. (2016) [93]	Discussed the essential signaling roles of reactive oxygen species in exercise, contributing to positive adaptations such as angiogenesis and improved muscle function.
Morales-Alamo and Calbet (2016) [94]	Concluded that antioxidant supplementation could potentially obstruct beneficial exercise adaptations by reducing the signaling role of reactive species, leading to a suboptimal redox state.
Theodorou et al. (2011) [95]	Found no effect of antioxidant supplementation on muscle performance and blood redox status adaptations to eccentric training, suggesting possible interference with beneficial exercise adaptations.
Radak et al. (2017) [96]	Linked excessive antioxidant supplementation to the potential progression of diseases like cancer and diabetes and even increased mortality, contributing to its negative reputation.
Sayin et al. (2014) [97]	Revealed significant interindividual variability in redox responses to oxidative stress, suggesting that baseline antioxidant levels influence the effectiveness of supplementation.
Margaritelis et al. (2014) [98]	Proposed that individual redox profiles could predict trainability and that antioxidant supplementation should be tailored based on these profiles to enhance exercise adaptations.
Margaritelis et al. (2017) [99]	Showed that antioxidant benefits, such as enhanced exercise adaptations, are more likely in individuals with specific deficiencies or high levels of oxidative stress.
Zeevi et al. (2015) [100]	Emphasized the need for personalized nutrition based on individual glycemic responses, highlighting the role of genetic factors in the effectiveness of nutritional interventions.
Wang and Hu (2018) [101]	Discussed the importance of precision nutrition in the management of type 2 diabetes, advocating for dietary adjustments based on individual genetic responses.
Corella et al. (2017) [102]	Underlined the influence of genetic factors on dietary responses, suggesting that nutritional genomics should guide diet customization to prevent cardiovascular diseases.
de Toro-Martín et al. (2017) [103]	Reviewed personalized nutritional approaches for metabolic syndrome, stressing the importance of considering individual dietary responses and metabolic profiles.
Gibney et al. (2013) [104]	Predicted the future direction of personalized nutrition, focusing on individual dietary needs based on specific phenotypic and genotypic profiles.
Hampl et al. (2004) [105]	Discussed the prevalence of vitamin C deficiency and its implications for health, suggesting targeted supplementation could correct specific nutrient deficiencies.
Nimni et al. (2007) [106]	Questioned the adequacy of dietary sulfur, pointing out its importance in human nutrition and the potential need for targeted supplementation in deficient populations.
Riedl et al. (2017) [107]	Introduced the concept of metabotyping for targeted nutrition, proposing that identifying metabolic profiles could guide more effective personalized nutrition strategies.
Maughan et al. (2018) [108]	Discussed the potential of a clinical tool to identify individual antioxidant deficiencies, proposing a threshold of F2-isoprostane for identifying candidates who might benefit from supplementation.
Paschalis et al. (2016) [109]	Highlighted significant interindividual variability in redox responses to exercise, showing that people with low levels of antioxidants initially perform worse physically but may benefit more from targeted supplementation.
Paschalis et al. (2018) [85]	Found that antioxidant supplementation can disrupt the GSH pathway, leading to broader disruptions in the antioxidative system, affecting systemic oxidative stress and physical performance.
Halliwell et al. (2015) [110]	Discussed how antioxidant enzymes play critical roles in regulating cellular signaling and energy metabolism, highlighting their selectivity and efficiency over other antioxidants.
Brigelius-Flohé et al. (2011) [111]	Explored the emerging concepts in the redox control of transcription factors, illustrating the complexity of redox regulation in cellular functions.
Forman et al. (2014) [112]	Provided an overview of mechanisms of redox signaling, emphasizing the nuanced role of antioxidants in cellular processes.
Margaritelis et al. (2016) [113]	Integrated reactive species into biological processes, particularly in exercise physiology, to explain variations in individual responses to physical activity.
Thomas et al. (2008) [114]	Discussed the chemical biology of nitric oxide, highlighting its role in cellular signaling related to redox states.
Cobley et al. (2015) [115]	Investigated the influence of vitamins C and E on redox signaling, suggesting potential interference with exercise adaptations due to antioxidant supplementation.
Perry and Hawley (2017) [116]	Discussed the molecular basis of exercise-induced skeletal muscle mitochondrial biogenesis, crucial for understanding the impact of antioxidants on this process.
Richter and Hargreaves (2013) [117]	Explored the role of GLUT4 in glucose uptake during exercise, emphasizing how reactive oxygen species can influence this pathway.
Merry and McConell (2009) [118]	Reviewed the influence of reactive oxygen and nitrogen species on skeletal muscle glucose uptake, underlining the complex role of these species in exercise.
Katz (2016) [119]	Highlighted the regulatory role of reactive oxygen species in glucose transport in skeletal muscle, indicating potential targets for antioxidant intervention.
Pinheiro et al. (2010) [120]	Examined how the redox state affects enzymes like creatine kinase, essential for energy production in muscle cells.
Koufen and Stark (2000) [121]	Studied the effects of oxidation on enzyme functionality, showing how an imbalanced redox state could impair energy metabolism.
Mitrea et al. (2018) [122] **	Examined the role of antioxidants in protecting against cosmic radiation-induced oxidative stress, relevant for individuals in high-altitude or intense solar exposure conditions.
Pfeiffer et al. (1999) [123] **	Investigated the effects of antioxidant supplementation at moderate altitudes, noting variable impacts on oxidative stress markers.
Paulsen et al. (2014) [124]	Demonstrated that vitamin C and E supplementation might inhibit beneficial physiological adaptations such as mitochondrial biogenesis during endurance training.
Yfanti et al. (2010) [125] **	Showed that supplementation with vitamins C and E did not enhance physical performance or adaptation to endurance training in healthy individuals.
Kesse-Guyot et al. (2011) [126]	Reported potential cognitive benefits of daily antioxidant vitamin and mineral supplementation, suggesting further evaluation in military contexts.

** Military context.

**Table 6:** Key Findings and Contributions on Nutritional Supplements.

Author(s) and Year	Key Findings and Contributions on Nutritional Supplements
Rhee et al. (2005) [127]	Discussed the complex roles of hydrogen peroxide and peroxiredoxins in intracellular signaling, illustrating the intricate regulation of antioxidants within cellular metabolism.
Azzi (2018) [128]	Highlighted the widespread deficiency in vitamin E intake among adults, emphasizing its crucial antioxidant role and the widespread neglect in achieving recommended dietary levels.
Maras et al. (2004) [129]	Reported that a significant percentage of the U.S. adult population does not meet the daily intake requirements for vitamin E, associating deficiency with various health issues.
Traber (2014) [130]	Explored the consequences of vitamin E deficiency, including severe neurological and metabolic dysfunctions, emphasizing the importance of adequate vitamin E intake.
Brigelius-Flohé et al. (2002) [131]	Reviewed the essential functions of vitamin E in preventing tissue damage from free radicals, especially in lipid-rich tissues, and discussed the reversibility of deficiency effects.
Galli et al. (2017) [132]	Investigated the broader impacts of vitamin E deficiency, linking severe cases to embryonic mortality and discussing persistent issues despite dietary corrections.
McDougall et al. (2017) [133]	Revealed that vitamin E deficiency during embryonic development leads to irreversible metabolic disruptions and increased risks of embryonic mortality, emphasizing the necessity of adequate vitamin E intake.
McDougall et al. (2017) [134]	Studied the irreversible damage caused by prolonged vitamin E deficiency in zebrafish models, showing lasting metabolic and developmental impacts.
Abner et al. (2011) [135]	Examined the potential risks associated with high-dose vitamin E supplementation, suggesting a balanced approach to avoid negative outcomes.
Miller et al. (2005) [136]	Conducted a meta-analysis indicating that excessive vitamin E supplementation may increase mortality, highlighting the need for caution in dosage.
Gill et al. (2014) [137]	Reported common vitamin D deficiencies in an Australian population, underlying the widespread issue across different populations.
Owens et al. (2015) [138]	Reviewed the critical roles of vitamin D in muscle function and immune response, particularly in athletes, stressing the importance of maintaining optimal levels.
Baeke et al. (2010) [139]	Discussed vitamin D as a modulator of the immune system, pointing out the broad health impacts of its deficiency.
Owens et al. (2015) [140]	Investigated the molecular mechanisms by which vitamin D supports muscle repair and hypertrophy, emphasizing its importance in physical rehabilitation.
Owens et al. (2017) [141]	Explored the benefits of high-dose vitamin D supplements for elite athletes, indicating its potential in optimizing physical performance.
Noble et al. (2014) [142]	Proposed a holistic approach to understanding the role of physiological processes in health and disease, advocating for a nuanced view of antioxidant supplementation.
Peternelj and Coombes (2011) [87]	Reviewed the mixed effects of antioxidant supplementation during exercise, suggesting a more tailored approach to its application based on individual needs.
Pingitore et al. (2015) [143] **	Emphasized the need for strategic use of antioxidants to maintain physical performance under oxidative stress, particularly in military contexts.
Lieberman (2010) [144] **	Highlighted the necessity of regular nutritional assessments to tailor dietary supplements to the specific needs of military personnel.
Attipoe et al. (2013) [145] **	Developed educational modules for military healthcare providers on dietary supplements, promoting informed and effective use.
Mantovani et al. (2006) [146] **	Demonstrated the potential benefits of integrating antioxidants in treatment regimens for cancer patients, showing improvements in nutritional status and quality of life.

** Military context.

## Data Availability

Not applicable.

## References

[1] Vaisman M., Ponce T., Barros T.R.D., Salerno V.P., Mainenti M.R.M. (2022). A systematic review of hormone levels, biomarkers of cellular injury and oxidative stress in multi-stressor military field training exercises. Arch. Endocrinol. Metab..

[2] Różański P., Jówko E., Tomczak A. (2020). Assessment of the Levels of Oxidative Stress, Muscle Damage, and Psychomotor Abilities of Special Force Soldiers during Military Survival Training. Int. J. Environ. Res. Public Health.

[3] van der Wal S.J., Vermetten E., Elbert G. (2020). Long-term development of post-traumatic stress symptoms and associated risk factors in military service members deployed to Afghanistan: Results from the PRISMO 10-year follow-up. Eur. Psychiatry.

[4] Chinoy E.D., Carey F.R., Kolaja C.A., Jacobson I.G., Cooper A.D., Markwald R.R. (2022). The bi-directional relationship between post-traumatic stress disorder and obstructive sleep apnea and/or insomnia in a large U.S. military cohort. Sleep Health.

[5] Böhm E.W., Buonfiglio F., Voigt A.M., Bachmann P., Safi T., Pfeiffer N., Gericke A. (2023). Oxidative stress in the eye and its role in the pathophysiology of ocular diseases. Redox Biol..

[6] Wang J., Li M., Geng Z., Khattak S., Ji X., Wu D., Dang Y. (2022). Role of oxidative stress in retinal disease and the early intervention strategies: A review. Oxid. Med. Cell. Longev..

[7] Ruan Y., Jiang S., Musayeva A., Gericke A. (2020). Oxidative stress and vascular dysfunction in the retina: Therapeutic strategies. Antioxidants.

[8] Shukla S., Mbingwa G., Khanna S., Dalal J., Sankhyan D., Malik A., Badhwar N. (2023). Environment and health hazards due to military metal pollution: A review. Environ. Nanotechnol. Monit. Manag..

[9] Zhang L., Chu J., Xia B., Xiong Z., Zhang S., Tang W. (2022). Health Effects of Particulate Uranium Exposure. Toxics.

[10] Darchini-Maragheh E., Balali-Mood M., Malaknezhad M., Mousavi S.R. (2018). Progressive delayed respiratory complications of sulfur mustard poisoning in 43 iranian veterans, three decades after exposure. Hum. Exp. Toxicol..

[11] Kiani B., Hashemi Amin F., Bagheri N., Bergquist R., Mohammadi A.A., Yousefi M., Faraji H., Roshandel G., Beirami S., Rahimzadeh H. (2021). Association between heavy metals and colon cancer: An ecological study based on geographical information systems in North-Eastern Iran. BMC Cancer.

[12] Manisalidis I., Stavropoulou E., Stavropoulos A., Bezirtzoglou E. (2020). Environmental and Health Impacts of Air Pollution: A Review. Front. Public Health.

[13] Hussain S., Khan M., Sheikh T.M.M., Mumtaz M.Z., Chohan T.A., Shamim S., Liu Y. (2022). Zinc Essentiality, Toxicity, and its Bacterial Bioremediation: A Comprehensive Insight. Front. Microbiol..

[14] Lisi V., Senesi G., Balbi C. (2023). Converging protective pathways: Exploring the linkage between physical exercise, extracellular vesicles and oxidative stress. Free Radic. Biol. Med..

[15] Fragala M.S., Cadore E.L., Dorgo S., Izquierdo M., Kraemer W.J., Peterson M.D., Ryan E.D. (2019). Resistance training for older adults: Position statement from the national strength and conditioning association. J. Strength Cond. Res..

[16] Jang W.Y., Kang D.O., Park Y., Lee J., Kim W., Choi J.Y., Roh S.Y., Jang Y., Park S.H., Kim W.S. (2020). Validation of FRIEND and ACSM equations for cardiorespiratory fitness: Comparison to direct measurement in CAD patients. J. Clin. Med..

[17] Helgerud J., Haglo H., Hoff J. (2022). Prediction of VO2max from submaximal exercise using the smartphone application myworkout GO: Validation study of a digital health method. JMIR Cardio.

[18] Schroeder E.C., Franke W.D., Sharp R.L., Lee D.C. (2019). Comparative effectiveness of aerobic, resistance, and combined training on cardiovascular disease risk factors: A randomized controlled trial. PLoS ONE.

[19] Haverkamp B.F., Wiersma R., Vertessen K., van Ewijk H., Oosterlaan J., Hartman E. (2020). Effects of physical activity interventions on cognitive outcomes and academic performance in adolescents and young adults: A meta-analysis. J. Sports Sci..

[20] Lake S.L., Guadagni V., Kendall K.D., Chadder M., Anderson T.J., Leigh R., Rawling J.M., Hogan D.B., Hill M.D., Poulin M.J. (2022). Aerobic exercise training in older men and women—Cerebrovascular responses to submaximal exercise: Results from the Brain in Motion study. Phys. Rep..

[21] Caplin A., Chen F.S., Beauchamp M.R., Puterman E. (2021). The effects of exercise intensity on the cortisol response to a subsequent acute psychosocial stressor. Psychoneuroendocrinology.

[22] Ryan B.J., Schleh M.W., Ahn C., Ludzki A.C., Gillen J.B., Varshney P., Van Pelt D.W., Pitchford L.M., Chenevert T.L., Gioscia-Ryan R.A. (2020). Moderate-intensity exercise and high-intensity interval training affect insulin sensitivity similarly in obese adults. J. Clin. Endocrinol. Metab..

[23] Bobinski F., Teixeira J.M., Sluka K.A., Santos A.R.S. (2018). Interleukin-4 mediates the analgesia produced by low-intensity exercise in mice with neuropathic pain. Pain.

[24] Otsuka Y., Yamada Y., Maeda A., Izumo T., Rogi T., Shibata H., Fukuda M., Arimitsu T., Miyamoto N., Hashimoto T. (2022). Effects of resistance training intensity on muscle quantity/quality in middle-aged and older people: A randomized controlled trial. J. Cachexia Sarcopenia Muscle.

[25] Sailani M.R., Halling J.F., Møller H.D., Lee H., Plomgaard P., Pilegaard H., Snyder M.P., Regenberg B. (2019). Lifelong physical activity is associated with promoter hypomethylation of genes involved in metabolism, myogenesis, contractile properties and oxidative stress resistance in aged human skeletal muscle. Sci. Rep..

[26] Hargreaves M., Spriet L.L. (2020). Skeletal muscle energy metabolism during exercise. Nat. Metab..

[27] Sorriento D., Di Vaia E., Iaccarino G. (2021). Physical exercise: A novel tool to protect mitochondrial health. Front. Physiol..

[28] Steinbacher P., Eckl P. (2015). Impact of oxidative stress on exercising skeletal muscle. Biomolecules.

[29] Powers S.K., Deminice R., Ozdemir M., Yoshihara T., Bomkamp M.P., Hyatt H. (2020). Exercise-induced oxidative stress: Friend or foe?. J. Sport Health Sci..

[30] Henríquez-Olguin C., Knudsen J.R., Raun S.H., Li Z., Dalbram E., Treebak J.T., Sylow L., Holmdahl R., Richter E.A., Jaimovich E. (2019). Cytosolic ROS production by NADPH oxidase 2 regulates muscle glucose uptake during exercise. Nat. Commun..

[31] Matta L., de Faria C.C., De Oliveira D.F., Andrade I.S., Lima-Junior N.C., Gregório B.M., Takiya C.M., Ferreira A.C.F., Nascimento J.H.M., de Carvalho D.P. (2022). Exercise improves redox homeostasis and mitochondrial function in white adipose tissue. Antioxidants.

[32] Baird L., Yamamoto M. (2020). The Molecular Mechanisms Regulating the KEAP1-NRF2 Pathway. Mol. Cell. Biol..

[33] Papanikolaou K., Jamurtas A.Z., Poulios A., Tsimeas P., Draganidis D., Margaritelis N.V., Baloyiannis I., Papadopoulos C., Sovatzidis A., Deli C.K. (2023). Skeletal muscle and erythrocyte redox status is associated with dietary cysteine intake and physical fitness in healthy young physically active men. Eur. J. Nutr..

[34] Pinto G., Militello R., Amoresano A., Modesti P.A., Modesti A., Luti S. (2022). Relationships between sex and adaptation to physical exercise in young athletes: A pilot study. Healthcare.

[35] Moreira-Reis A., Maté-Muñoz J.L., Hernández-Lougedo J., Vilches-Sáez S., Benet M., García-Fernández P., Pleguezuelos E., Carbonell T., Alva N., Garnacho-Castaño M.V. (2022). Aerobic dance on an air dissipation platform improves cardiorespiratory, muscular and cellular fitness in the overweight and obese elderly. Biology.

[36] Gonzalez-Gil A.M., Elizondo-Montemayor L. (2020). The role of exercise in the interplay between myokines, hepatokines, osteokines, adipokines, and modulation of inflammation for energy substrate redistribution and fat mass loss: A review. Nutrients.

[37] Chow L.S., Gerszten R.E., Taylor J.M., Pedersen B.K., Van Praag H., Trappe S., Febbraio M.A., Galis Z.S., Gao Y., Haus J.M. (2022). Exerkines in health, resilience and disease. Nat. Rev. Endocrinol..

[38] Thyfault J.P., Bergouignan A. (2020). Exercise and metabolic health: Beyond skeletal muscle. Diabetologia.

[39] Yáñez-Mó M., Siljander P.R.M., Andreu Z., Bedina Zavec A., Borràs F.E., Buzas E.I., Buzas K., Casal E., Cappello F., Carvalho J. (2015). Biological properties of extracellular vesicles and their physiological functions. J. Extracell. Vesicles.

[40] Gruenberg J. (2020). Life in the lumen: The multivesicular endosome. Traffic.

[41] Whitham M., Parker B.L., Friedrichsen M., Hingst J.R., Hjorth M., Hughes W.E., Egan C.L., Cron L., Watt K.I., Kuchel R.P. (2018). Extracellular vesicles provide a means for tissue crosstalk during exercise. Cell Metabol..

[42] Alberro A., Iparraguirre L., Fernandes A., Otaegui D. (2021). Extracellular vesicles in blood: Sources, effects, and applications. Int. J. Mol. Sci..

[43] Hink U., Li H., Mollnau H., Oelze M., Matheis E., Hartmann M., Skatchkov M., Thaiss F., Stahl R.A., Warnholtz A. (2001). Mechanisms underlying endothelial dysfunction in diabetes mellitus. Circ. Res..

[44] Szivak T.K., Kraemer W.J. (2015). Physiological Readiness and Resilience: Pillars of Military Preparedness. J. Strength Cond. Res..

[45] Chang C.F., Wu Y.C., Lai C.H., Chen P.C., Guo Y.L. (2023). Effects of physical fitness training on metabolic syndrome among military personnel in Taiwan. BMJ Mil. Health.

[46] Hedlund E., Börjesson M., Österberg J. (2015). Team Learning in a Multinational Military Staff Exercise. Small Group. Res..

[47] Manning F.J., Fullerton T.D. (1988). Health and well-being in highly cohesive units of the U.S. Army. J. Appl. Soc. Psychol..

[48] Adler A.B., Williams J., McGurk D., Moss A., Bliese P.D. (2015). Resilience training with soldiers during basic combat training: Randomisation by platoon. Appl. Psychol. Health Well Being.

[49] Kyröläinen H., Pihlainen K., Vaara J.P., Ojanen T., Santtila M. (2018). Optimising training adaptations and performance in military environment. J. Sci. Med. Sport.

[50] Vantarakis A., Chatzinikolaou A., Avloniti A., Vezos N., Douroudos I.I., Draganidis D., Jamurtas A.Z., Kambas A., Kalligeros S., Fatouros I.G. (2017). A 2-Month Linear Periodized Resistance Exercise Training Improved Musculoskeletal Fitness and Specific Conditioning of Navy Cadets. J. Strength Cond. Res..

[51] Jovanov E., Lords A.O., Raskovic D., Cox P.G., Adhami R., Andrasik F. (2003). Stress monitoring using a distributed wireless intelligent sensor system. IEEE Eng. Med. Biol. Mag..

[52] Tanskanen M.M., Uusitalo A.L., Kinnunen H., Häkkinen K., Kyröläinen H., Atalay M. (2011). Association of military training with oxidative stress and overreaching. Med. Sci. Sports Exerc..

[53] Hall K.S., Morey M.C., Beckham J.C., Bosworth H.B., Sloane R., Pieper C.F., Pebole M.M. (2020). Warrior Wellness: A Randomized Controlled Pilot Trial of the Effects of Exercise on Physical Function and Clinical Health Risk Factors in Older Military Veterans With PTSD. J. Gerontol. A Biol. Sci. Med. Sci..

[54] Allen B.G., Bhatia S.K., Buatti J.M., Brandt K.E., Lindholm K.E., Button A.M., Szweda L.I., Smith B.J., Spitz D.R., Fath M.A. (2013). Ketogenic diets enhance oxidative stress and radio-chemo-therapy responses in lung cancer xenografts. Clin. Cancer Res..

[55] Atakan M.M., Li Y., Koşar Ş.N., Turnagöl H.H., Yan X. (2021). Evidence-Based effects of high-intensity interval training on exercise capacity and health: A review with historical perspective. Int. J. Environ. Res. Public Health.

[56] Pinto A., Bonucci A., Maggi E., Corsi M., Businaro R. (2018). Anti-oxidant and anti-inflammatory activity of ketogenic diet: New perspectives for neuroprotection in Alzheimer’s disease. Antioxidants.

[57] Reuter S., Gupta S.C., Chaturvedi M.M., Aggarwal B.B. (2010). Oxidative stress, inflammation, and cancer: How are they linked?. Free Radic. Biol. Med..

[58] Kodydkova J., Vavrova L., Stankova B., Macasek J., Krechler T., Zak A. (2013). Antioxidant status and oxidative stress markers in pancreatic cancer and chronic pancreatitis. Pancreas.

[59] Lin W., Karin M. (2007). A cytokine-mediated link between innate immunity, inflammation, and cancer. J. Clin. Investig..

[60] Ostan R., Lanzarini C., Pini E., Scurti M., Vianello D., Bertarelli C., Fabbri C., Izzi M., Palmas G., Biondi F. (2015). Inflammaging and Cancer: A challenge for the mediterranean diet. Nutrients.

[61] Susan P. (2005). Modulation of oxidative stress and mitochondrial function by the ketogenic diet. Bone.

[62] Poff A.M., Ari C., Seyfried T.N., D’Agostino D.P. (2013). The ketogenic diet and hyperbaric oxygen therapy prolong survival in mice with systemic metastatic cancer. PLoS ONE.

[63] Nazarewicz R.R., Ziolkowski W., Vaccaro P.S., Ghafourifar P. (2007). Effect of short-term ketogenic diet on redox status of human blood. Rejuvenation Res..

[64] Sullivan P.G., Rippy N.A., Dorenbos K., Concepcion R.C., Agarwal A.K., Rho J.M. (2004). The ketogenic diet increases mitochondrial uncoupling protein levels and activity. Ann. Neurol..

[65] Tieu K., Perier C., Caspersen C., Teismann P., Wu D.C., Yan S.D., Naini A., Vila M., Jackson-Lewis V., Ramasamy R. (2003). D-β-Hydroxybutyrate rescues mitochondrial respiration and mitigates features of Parkinson disease. J. Clin. Investig..

[66] Chorley B.N., Campbell M.R., Wang X., Karaca M., Sambandan D., Bangura F., Xue P., Pi J., Kleeberger S.R., Bell D.A. (2012). Identification of novel NRF2-regulated genes by ChiP-Seq: Influence on retinoid X receptor alpha. Nucleic Acids Res..

[67] Shimazu T., Hirschey M.D., Newman J., He W., Le Moan N., Grueter C.A., Lim H., Laura R., Stevens R.D., Newgard C.B. (2013). Supression of oxidative stress and β-OHB as endogenous histone deactetylase. Science.

[68] Yang Y., Sauve A.A. (2016). NAD+ metabolism: Bioenergetics, signaling and manipulation for therapy. Biochim. Biophys. Acta Proteins Proteom..

[69] Jarrett S.G., Milder J.B., Liang L.P., Patel M. (2008). The ketogenic diet increases mitochondrial glutathione levels. J. Neurochem..

[70] Elamin M., Ruskin D.N., Masino S.A., Sacchetti P. (2018). Ketogenic diet modulates NAD+-dependent enzymes and reduces DNA damage in hippocampus. Front. Cell. Neurosci..

[71] Stafford P., Abdelwahab M.G., Kim D.Y., Preul M.C., Rho J.M., Scheck A.C. (2010). The ketogenic diet reverses gene expression patterns and reduces reactive oxygen species levels when used as an adjuvant therapy for glioma. Nutr. Metab..

[72] Alhamzah S.A., Gatar O.M., Alruwaili N.W. (2023). Effects of ketogenic diet on oxidative stress and cancer: A literature review. Adv. Cancer Biol. Metastasis.

[73] Xu T., Cao L., Zhou M., Chen W. (2020). The influencing factors of Upin peroxination in the body. Public Health Prev. Med..

[74] Kong W., Jiang T., Ning Y., Guo Y., Liu H., Lyu X., Li M. (2022). Dietary diversity, diet quality, and oxidative stress in older adults. Geriatr. Nurs..

[75] Aleksandrova K., Koelman L., Rodrigues C.E. (2021). Dietary patterns and biomarkers of oxidative stress and inflammation: A systematic review of observational and intervention studies. Redox Biol..

[76] Zhang Q., Chen X., Liu Z., Varma D.S., Wan R., Zhao S. (2017). Diet diversity and nutritional status among adults in southwest China. PLoS ONE.

[77] Chai S.C., Davis K., Zhang Z., Zha L., Kirschner K.F. (2019). Effects of Tart Cherry Juice on Biomarkers of Inflammation and Oxidative Stress in Older Adults. Nutrients.

[78] Newman J.C., Covarrubias A.J., Zhao M., Yu X., Gut P., Ng C.P., Huang Y., Haldar S., Verdin E. (2017). Ketogenic Diet Reduces Midlife Mortality and Improves Memory in Aging Mice. Cell Metab..

[79] Greco T., Glenn T.C., Hovda D.A., Prins M.L. (2016). Ketogenic diet decreases oxidative stress and improves mitochondrial respiratory complex activity. J. Cereb. Blood Flow Metab..

[80] LaFountain R.A., Miller V.J., Barnhart E.C., Hyde P.N., Crabtree C.D., McSwiney F.T., Beeler M.K., Buga A., Sapper T.N., Short J.A. (2019). Extended Ketogenic Diet and Physical Training Intervention in Military Personnel. Mil. Med..

[81] Volek J.S., LaFountain R.A., Dituro P. (2019). Extended Ketogenic Diet and Physical Training Intervention in Military Personnel. Mil. Med..

[82] Tan B.L., Norhaizan M.E. (2019). Effect of High-Fat Diets on Oxidative Stress, Cellular Inflammatory Response and Cognitive Function. Nutrients.

[83] Nikolaidis M.G., Kyparos A., Spanou C., Paschalis V., Theodorou A.A., Vrabas I.S. (2012). Redox biology of exercise: An integrative and comparative consideration of some overlooked issues. J. Exp. Biol..

[84] Merry T.L., Ristow M. (2016). Do antioxidant supplements interfere with skeletal muscle adaptation to exercise training?. J. Physiol..

[85] Paschalis V., Theodorou A.A., Margaritelis N.V., Kyparos A., Nikolaidis M.G. (2018). N-acetylcysteine supplementation increases exercise performance and reduces oxidative stress only in individuals with low levels of glutathione. Free Radic. Biol. Med..

[86] Michailidis Y., Karagounis L.G., Terzis G., Jamurtas A.Z., Spengos K., Tsoukas D., Chatzinikolaou A., Mandalidis D., Stefanetti R.J., Papassotiriou I. (2013). Thiol-based antioxidant supplementation alters human skeletal muscle signaling and attenuates its inflammatory response and recovery after intense eccentric exercise. Am. J. Clin. Nutr..

[87] Peternelj T.T., Coombes J.S. (2011). Antioxidant supplementation during exercise training: Beneficial or detrimental?. Sports Med..

[88] Pļaviņa L., Koļesova O., Eglīte J., Čakstiņš A., Cakstina S., Koļesovs A. (2021). Antioxidative system capacity after a 10-day-long intensive training course and one-month-long recovery in military cadets. Phys. Act. Rev..

[89] Knapik J.J., Austin K.G., Farina E.K., Lieberman H.R. (2018). Dietary Supplement Use in a Large, Representative Sample of the US Armed Forces. J. Acad. Nutr. Diet..

[90] Macedo R.C., Vieira A., Marin D.P., Otton R. (2015). Effects of chronic resveratrol supplementation in military firefighters undergo a physical fitness test--a placebo-controlled, double blind study. Chem. Biol. Interact..

[91] Meydani S.N., Meydani M., Blumberg J.B., Leka L.S., Pedrosa M., Diamond R., Schaefer E.J. (1998). Assessment of the safety of supplementation with different amounts of vitamin E in healthy older adults. Am. J. Clin. Nutr..

[92] Margaritelis N.V., Paschalis V., Theodorou A.A., Kyparos A., Nikolaidis M.G. (2018). Antioxidants in Personalized Nutrition and Exercise. Adv. Nutr..

[93] Mason S.A., Morrison D., McConell G.K., Wadley G.D. (2016). Muscle redox signalling pathways in exercise: Role of antioxidants. Free Radic. Biol. Med..

[94] Morales-Alamo D., Calbet J.A. (2016). AMPK signaling in skeletal muscle during exercise: Role of reactive oxygen and nitrogen species. Free Radic. Biol. Med..

[95] Theodorou A.A., Nikolaidis M.G., Paschalis V., Koutsias S., Panayiotou G., Fatouros I.G., Koutedakis Y., Jamurtas A.Z. (2011). No effect of antioxidant supplementation on muscle performance and blood redox status adaptations to eccentric training. Am. J. Clin. Nutr..

[96] Radak Z., Ishihara K., Tekus E., Varga C., Posa A., Balogh L., Boldogh I., Koltai E. (2017). Exercise, oxidants, and antioxidants change the shape of the bell-shaped hormesis curve. Redox Biol..

[97] Sayin V.I., Ibrahim M.X., Larsson E., Nilsson J.A., Lindahl P., Bergo M.O. (2014). Antioxidants accelerate lung cancer progression in mice. Sci. Transl. Med..

[98] Margaritelis N.V., Kyparos A., Paschalis V., Theodorou A.A., Panayiotou G., Zafeiridis A., Dipla K., Nikolaidis M.G., Vrabas I.S. (2014). Reductive stress after exercise: The issue of redox individuality. Redox Biol..

[99] Margaritelis N.V., Theodorou A.A., Paschalis V., Veskoukis A.S., Dipla K., Zafeiridis A., Panayiotou G., Vrabas I.S., Kyparos A., Nikolaidis M.G. (2018). Adaptations to endurance training depend on exercise-induced oxidative stress: Exploiting redox inter-individual variability. Acta Physiol..

[100] Zeevi D., Korem T., Zmora N., Israeli D., Rothschild D., Weinberger A., Ben-Yacov O., Lador D., Avnit-Sagi T., Lotan-Pompan M. (2015). Personalized nutrition by prediction of glycemic responses. Cell.

[101] Wang D.D., Hu F.B. (2018). Precision nutrition for prevention and management of type 2 diabetes. Lancet Diabetes Endocrinol..

[102] Corella D., Coltell O., Mattingley G., Sorlí J.V., Ordovas J.M. (2017). Utilizing nutritional genomics to tailor diets for the prevention of cardiovascular disease: A guide for upcoming studies and implementations. Expert Rev. Mol. Diagn..

[103] de Toro-Martín J., Arsenault B.J., Després J.P., Vohl M.C. (2017). Precision nutrition: A review of personalized nutritional approaches for the prevention and management of metabolic syndrome. Nutrients.

[104] Gibney M.J., Walsh M.C. (2013). The future direction of personalised nutrition: My diet, my phenotype, my genes. Proc. Nutr. Soc..

[105] Hampl J.S., Taylor C.A., Johnston C.S. (2004). Vitamin C deficiency and depletion in the United States: The Third National Health and Nutrition Examination Survey, 1988 to 1994. Am. J. Public Health.

[106] Nimni M.E., Han B., Cordoba F. (2007). Are we getting enough sulfur in our diet?. Nutr. Metab..

[107] Riedl A., Gieger C., Hauner H., Daniel H., Linseisen J. (2017). Metabotyping and its application in targeted nutrition: An overview. Br. J. Nutr..

[108] Maughan R.J., Burke L.M., Dvorak J., Larson-Meyer D.E., Peeling P., Phillips S.M., Rawson E.S., Walsh N.P., Garthe I., Geyer H. (2018). IOC consensus statement: Dietary supplements and the high-performance athlete. Int. J. Sport Nutr. Exerc. Metab..

[109] Paschalis V., Theodorou A.A., Kyparos A., Dipla K., Zafeiridis A., Panayiotou G., Vrabas I.S., Nikolaidis M.G. (2016). Low vitamin C values are linked with decreased physical performance and increased oxidative stress: Reversal by vitamin C supplementation. Eur. J. Nutr..

[110] Halliwell B., Gutteridge J. (2015). Free Radicals in Biology and Medicine.

[111] Brigelius-Flohé R., Flohé L. (2011). Basic principles and emerging concepts in the redox control of transcription factors. Antioxid. Redox Signal..

[112] Forman H.J., Ursini F., Maiorino M. (2014). An overview of mechanisms of redox signaling. J. Mol. Cell. Cardiol..

[113] Margaritelis N.V., Cobley J.N., Paschalis V., Veskoukis A.S., Theodorou A.A., Kyparos A., Nikolaidis M.G. (2016). Principles for integrating reactive species into in vivo biological processes: Examples from exercise physiology. Cell. Signal..

[114] Thomas D.D., Ridnour L.A., Isenberg J.S., Flores-Santana W., Switzer C.H., Donzelli S., Hussain P., Vecoli C., Paolocci N., Ambs S. (2008). The chemical biology of nitric oxide: Implications in cellular signaling. Free Radic. Biol. Med..

[115] Cobley J.N., McHardy H., Morton J.P., Nikolaidis M.G., Close G.L. (2015). Influence of vitamin C and vitamin E on redox signaling: Implications for exercise adaptations. Free Radic. Biol. Med..

[116] Perry C.G.R., Hawley J.A. (2018). Molecular basis of exercise-induced skeletal muscle mitochondrial biogenesis: Historical advances, current knowledge, and future challenges. Cold Spring Harb. Perspect. Med..

[117] Richter E.A., Hargreaves M. (2013). Exercise, GLUT4, and skeletal muscle glucose uptake. Physiol. Rev..

[118] Merry T.L., McConell G.K. (2009). Skeletal muscle glucose uptake during exercise: A focus on reactive oxygen species and nitric oxide signaling. IUBMB Life.

[119] Katz A. (2016). Role of reactive oxygen species in regulation of glucose transport in skeletal muscle during exercise. J. Physiol..

[120] Pinheiro C.H., Silveira L.R., Nachbar R.T., Vitzel K.F., Curi R. (2010). Regulation of glycolysis and expression of glucose metabolism-related genes by reactive oxygen species in contracting skeletal muscle cells. Free Radic. Biol. Med..

[121] Koufen P., Stark G. (2000). Free radical induced inactivation of creatine kinase: Sites of interaction, protection, and recovery. Biochim. Biophys. Acta.

[122] Mitrea D.R., Mortazavi Moshkenani H., Hoteiuc O.A., Bidian C., Toader A.M., Clichici S. (2018). Antioxidant protection against cosmic radiation-induced oxidative stress at commercial flight altitude. J. Physiol. Pharmacol..

[123] Pfeiffer J.M., Askew E.W., Roberts D.E., Wood S.M., Benson J.E., Johnson S.C., Freedman M.S. (1999). Effect of antioxidant supplementation on urine and blood markers of oxidative stress during extended moderate-altitude training. Wilderness Environ. Med..

[124] Paulsen G., Cumming K.T., Holden G., Hallén J., Rønnestad B.R., Sveen O., Skaug A., Paur I., Bastani N.E., Østgaard H.N. (2014). Vitamin C and E supplementation hampers cellular adaptation to endurance training in humans: A double-blind, randomised, controlled trial. J. Physiol..

[125] Yfanti C., Åkerström T., Nielsen S., Nielsen A., Mounier R., Mortensen O., Lykkesfeldt J., Rose A., Fischer C., Pedersen B. (2010). Antioxidant supplementation does not alter endurance training adaptation. Med. Sci. Sports Exerc..

[126] Kesse-Guyot E., Fezeu L., Jeandel C., Ferry M., Andreeva V., Amieva H., Hercberg S., Galan P. (2011). French adults’ cognitive performance after daily supplementation with antioxidant vitamins and minerals at nutritional doses: A post hoc analysis of the Supplementation in Vitamins and Mineral Antioxidants (SU.VI.MAX) trial. Am. J. Clin. Nutr..

[127] Rhee S.G., Kang S.W., Jeong W., Chang T.S., Yang K.S., Woo H.A. (2005). Intracellular messenger function of hydrogen peroxide and its regulation by peroxiredoxins. Curr. Opin. Cell Biol..

[128] Azzi A. (2018). Many tocopherols, one vitamin, E. Mol. Asp. Med..

[129] Maras J.E., Bermudez O.I., Qiao N., Bakun P.J., Boody-Alter E.L., Tucker K.L. (2004). Intake of alpha-tocopherol is limited among US adults. J. Am. Diet. Assoc..

[130] Traber M.G. (2014). Vitamin E inadequacy in humans: Causes and consequences. Adv. Nutr..

[131] Brigelius-Flohé R., Kelly F.J., Salonen J.T., Neuzil J., Zingg J.M., Azzi A. (2002). The European perspective on vitamin E: Current knowledge and future research. Am. J. Clin. Nutr..

[132] Galli F., Azzi A., Birringer M., Cook-Mills J.M., Eggersdorfer M., Frank J., Cruciani G., Lorkowski S., Özer N.K. (2017). Vitamin E: Emerging aspects and new directions. Free Radic. Biol. Med..

[133] McDougall M., Choi J., Kim H.K., Bobe G., Stevens J.F., Cadenas E., Tanguay R., Traber M.G. (2017). Lethal dysregulation of energy metabolism during embryonic vitamin E deficiency. Free Radic. Biol. Med..

[134] McDougall M., Choi J., Truong L., Tanguay R., Traber M.G. (2017). Vitamin E deficiency during embryogenesis in zebrafish causes lasting metabolic and cognitive impairments despite refeeding adequate diets. Free Radic. Biol. Med..

[135] Abner E.L., Schmitt F.A., Mendiondo M.S., Marcum L., Kryscio R.J. (2011). Vitamin E and all-cause mortality: A meta-analysis. Curr. Aging Sci..

[136] Miller E.R., Pastor-Barriuso R., Dalal D., Riemersma R.A., Appel L.J., Guallar E. (2005). Meta-analysis: High-dosage vitamin E supplementation may increase all-cause mortality. Ann. Intern. Med..

[137] Gill T.K., Hill C.L., Shanahan E.M., Taylor A.W., Appleton S.L., Grant J.F., Shi Z., Dal Grande E., Price K., Adams R.J. (2014). Vitamin D levels in an Australian population. BMC Public Health.

[138] Owens D.J., Fraser W.D., Close G.L. (2015). Vitamin D and the athlete: Emerging insights. Eur. J. Sport Sci..

[139] Baeke F., Takiishi T., Korf H., Gysemans C., Mathieu C. (2010). Vitamin D: Modulator of the immune system. Curr. Opin. Pharmacol..

[140] Owens D.J., Sharples A.P., Polydorou I., Alwan N., Donovan T., Tang J., Fraser W.D., Cooper R.G., Morton J.P., Stewart C. (2015). A systems-based investigation into vitamin D and skeletal muscle repair, regeneration, and hypertrophy. Am. J. Physiol. Endocrinol. Metab..

[141] Owens D.J., Tang J.C., Bradley W.J., Sparks A.S., Fraser W.D., Morton J.P., Close G.L. (2017). Efficacy of high-dose vitamin D supplements for elite athletes. Med. Sci. Sports Exerc..

[142] Noble D., Jablonka E., Joyner M.J., Müller G.B., Omholt S.W. (2014). Evolution evolves: Physiology returns to centre stage. J. Physiol..

[143] Pingitore A., Lima G.P., Mastorci F., Quinones A., Iervasi G., Vassalle C. (2015). Exercise and oxidative stress: Potential effects of antioxidant dietary strategies in sports. Nutrition.

[144] Lieberman H.R., Stavinoha T.B., McGraw S.M., White A., Hadden L.S., Marriott B.P. (2010). Use of dietary supplements among active-duty US Army soldiers. Am. J. Clin. Nutr..

[145] Attipoe S., Costello R., Kohlmeier M., Deuster P. (2013). Dietary supplement education for the military: An education module for healthcare providers. FASEB J..

[146] Mantovani G., Macciò A., Madeddu C., Gramignano G., Lusso M.R., Serpe R., Massa E., Astara G., Deiana L. (2006). A phase II study with antioxidants, both in the diet and supplemented, pharmaconutritional support, progestagen, and anti-cyclooxygenase-2 showing efficacy and safety in patients with cancer-related anorexia/cachexia and oxidative stress. Cancer Epidemiol. Biomark. Prev..

[147] Weeks S.R., McAuliffe C.L., Durussel D., Pasquina P.F. (2010). Physiological and psychological fatigue in extreme conditions: The military example. PM R.

[148] Cox P.J., Kirk T., Ashmore T., Willerton K., Evans R., Smith A., Murray A.J., Stubbs B., West J., McLure S.W. (2016). Nutritional Ketosis Alters Fuel Preference and Thereby Endurance Performance in Athletes. Cell Metab..

[149] Li R.J., Liu Y., Liu H.Q., Li J. (2020). Ketogenic diets and protective mechanisms in epilepsy, metabolic disorders, cancer, neuronal loss, and muscle and nerve degeneration. J. Food Biochem..

[150] Parastouei K., Rostami H., Velikova T., Alipour M. (2018). Role of nutritional supplements in military personnel: A review article. Int. J. Biomed. Public Health.

